# LCAT3, a novel m6A-regulated long non-coding RNA, plays an oncogenic role in lung cancer via binding with FUBP1 to activate c-MYC

**DOI:** 10.1186/s13045-021-01123-0

**Published:** 2021-07-17

**Authors:** Xinyi Qian, Juze Yang, Qiongzi Qiu, Xufan Li, Chengxi Jiang, Jia Li, Liangliang Dong, Kejing Ying, Bingjian Lu, Enguo Chen, Pengyuan Liu, Yan Lu

**Affiliations:** 1 Center for Uterine Cancer Diagnosis and Therapy Research of Zhejiang Province, Women’s Reproductive Health Key Laboratory of Zhejiang Province, Department of Gynecologic Oncology, Women’s Hospital and Institute of Translational Medicine, Zhejiang University School of Medicine, Hangzhou, 310006 Zhejiang China; 2 Department of Respiratory Medicine, Sir Run Run Shaw Hospital and Institute of Translational Medicine, Zhejiang University School of Medicine, Hangzhou, 310016 Zhejiang China; 3 Chemical Biology Research Center, College of Pharmaceutical Sciences, Wenzhou Medical University, Wenzhou, 325000 Zhejiang China; 4 Cancer Center, Zhejiang University, Hangzhou, 310013 Zhejiang China; 5 Department of Physiology and Center of Systems Molecular Medicine, Medical College of Wisconsin, Milwaukee, 53226 WI USA

**Keywords:** LCAT3, Lung cancer, Long noncoding RNAs, FUBP1, MYC, N6-methyladenosine

## Abstract

**Background:**

Long non-coding RNAs (lncRNAs) are important epigenetic regulators, which play critical roles in diverse physiological and pathological processes. However, the regulatory mechanism of lncRNAs in lung carcinogenesis remains elusive. Here, we characterized a novel oncogenic lncRNA, designated as Lung Cancer Associated Transcript 3 (LCAT3).

**Methods:**

We predicted and validated LCAT3 by analyzing RNA-sequencing (RNA-seq) data of lung cancer tissues from TCGA. Methylated RNA immunoprecipitation was performed to assess m6A modification on LCAT3. The LCAT3-FUBP1-MYC axis was assessed by dual-luciferase reporter, RNA immunoprecipitation and Chromatin immunoprecipitation assays. Signaling pathways altered by LCAT3 knockdown were identified using RNA-seq. Furthermore, the mechanism of LCAT3 was investigated using loss-of-function and gain-of-function assays in vivo and in vitro.

**Results:**

LCAT3 was found to be up-regulated in lung adenocarcinomas (LUAD), and its over-expression was associated with the poor prognosis of LUAD patients. LCAT3 upregulation is attributable to N6-methyladenosine (m6A) modification mediated by methyltransferase like 3 (METTL3), leading to LCAT3 stabilization. Biologically, loss-of-function assays revealed that LCAT3 knockdown significantly suppressed lung cancer cell proliferation, migration and invasion in vitro, and inhibited tumor growth and metastasis in vivo. LCAT3 knockdown induced cell cycle arrest at the G1 phase. Mechanistically, LCAT3 recruited Far Upstream Element Binding Protein 1 (FUBP1) to the MYC far-upstream element (FUSE) sequence, thereby activating MYC transcription to promote proliferation, survival, invasion and metastasis of lung cancer cells.

**Conclusions:**

Taken together, we identified and characterized LCAT3 as a novel oncogenic lncRNA in the lung, and validated the LCAT3-FUBP1-MYC axis as a potential therapeutic target for LUAD.

**Supplementary Information:**

The online version contains supplementary material available at 10.1186/s13045-021-01123-0.

## Background

Lung cancer is the most commonly diagnosed cancer and the leading cause of cancer-related death worldwide [1]. It is classified into small cell lung cancer (SCLC) and non-small cell lung cancer (NSCLC). While NSCLC constitutes about 85% of all lung cancer cases, lung adenocarcinomas (LUAD) are the most common subtype, accounting for more than 40% of cases. Despite recent advancement in targeted therapies and immunotherapies, the 5-year survival rate of lung cancer patients remains dismal and varies from 4 to 17%, depending on tumor stage and regional lymph node status [2]. Identification of early diagnostic and therapeutic biomarkers critically involved in lung cancer progression is, therefore, of great clinical significance.

Long non-coding RNAs (lncRNAs) are a subclass of transcripts that are longer than 200 nucleotides and that have limited or no protein coding potential. In recent years, lncRNAs are emerging as important regulators in gene expression networks via their control of nuclear architecture and transcription in the nucleus, and via their modulation of mRNA stability, translation and post-translational modifications in the cytoplasm [3–8]. Several lncRNAs, such as Xist [9], TUG1 [10], lnc-IGFBP4-1 [11], LINC00336 [12] and LINC01123 [13], have been reported to be aberrantly expressed in lung cancer and shown to regulate lung tumorigenesis and progression. For instance, TUG1 binds with the enhancer of EZH2 and regulates the expression of LIMK2b, thereby promoting the cell growth and chemo-resistance of SCLC [10]. In addition, LINC00336 serves as an endogenous sponge of miR6852 to regulate the expression of cystathionine-β-synthase (CBS) and ferroptosis in lung cancer [12].

In the present study, we reported the identification of a novel lncRNA, designated as Lung Cancer Associated Transcript 3 (LCAT3), that is remarkably upregulated in lung cancer tissues with positive association of poor prognosis. LCAT3 upregulation is attributable to N6-methyladenosine (m6A) modification mediated by methyltransferase like 3 (METTL3), leading to LCAT3 stabilization. Functional assays demonstrated that LCAT3 promotes proliferation, survival, migration and metastasis of lung cancer cells both in vitro cell culture and in vivo xenograft models. Mechanistic analysis reveals that LCAT3 interacted with Far Upstream Element Binding Protein 1 (FUBP1), leading to transactivation of c-MYC expression. Taken together, our results demonstrate that LCAT3 plays a pivotal role in enhancing malignant phenotypes of lung cancer cells, which may serve as an attractive therapeutic target for lung cancer.

## Methods

### Cell culture

The human LUAD cell lines (A549, Calu1 and Hop62) and a human embryonic kidney cell line (HEK-293T) were purchased from the American Type Culture Collection (ATCC, Manassas, VA, USA). A549, Calu1 and Hop62 cells were cultured in Roswell Park Memorial Institute 1640 Media (RPMI 1640; Gibco, Carlsbad, CA, USA). HEK-293T cells were cultured in Dulbecco's Modified Eagle Medium (DMEM; Gibco). All medium was supplemented with 10% fetal bovine serum (FBS; Gibco) and 1% penicillin/streptomycin (Gibco). All cell lines were cultured in a humidified atmosphere with 5% CO_2_ and at 37 °C.

### RNA isolation, reverse transcription, and real-time PCR

Total RNA from cell culture or tissue samples was isolated using TRIzol reagent (Invitrogen, Carlsbad, CA, USA) following the manufacturer’s instructions. 1 µg of total RNA was reverse transcribed using HiScript II Q RT SuperMix for qPCR (+gDNA wiper) (Vazyme, Nanjing, China). Real-time PCR was performed on the cDNA using ChamQ Universal SYBR qPCR Master Mix (Vazyme). β-Actin was used as an endogenous control for normalization. All primer sequences are listed in Additional file 1: Table S1. The relative fold changes in expression were analyzed using the 2^−△△CT^ method.

### Cytoplasmic and nuclear RNA isolation

A PARIS Kit (Life Technologies, CA, USA) was used to isolate the cytoplasmic and nuclear fraction following the manufacturer's protocol. Briefly, cells were lysed using a cell fractionation buffer and incubated on ice for 10 min. By centrifuging at 500 g for 5 min, the supernatant was collected as the cytoplasmic fraction, and the remaining nuclear pellets were lysed with cell disruption buffer as the nuclear fraction. The cytoplasmic and nuclear RNA was then extracted and analyzed by qRT-PCR.

### Rapid amplification of cDNA ends (RACE) assay

RACE PCR products were obtained using a SMARTer RACE 5′/3′ Kit (Takara, CA, USA) and separated on a 1.2% agarose gel. The gel products were extracted using a gel extraction kit (Omega, Norcross, GA, USA), cloned into a T5-Zero vector (Transgen, Beijing, China) and sequenced. The specific 3′ RACE and 5′ RACE primers are listed in Additional file 1: Table S1.

### Plasmid construction and cell transfection

For knockdown of LCAT3 and FUBP1, specific small interference RNAs (siRNAs) synthesized from GenePharma (Shanghai, China) were transfected into cells with GeneMute™ reagent (SignaGen™ Laboratories, Rockville, MD, USA) at a final concentration of 50 nM. To stably knockdown LCAT3 in lung cancer cells, two small hairpin RNAs (shRNAs) were synthesized and cloned into a lentiviral vector pLKO.1. For the overexpression assay, a full-length human LCAT3 sequence was obtained by PCR and subcloned into the pCDNA 3.1 vector by Lipofectamine 3000 (Invitrogen) to establish cells that stably overexpressed LCAT3. For the METTL3 knockout assay, two single guide RNAs (sgRNAs) were designed using the guide design tool (http://crispr.mit.edu/) and cloned into lenti-gRNA-puro. The lentivirus was collected 48 h after co-transfection of the lenti-gRNA, pCMV-vsvg, and pSPAX2 vector into HEK293T cells using Lipofectamine 3000 transfection reagent. Calu1 cells were infected with filtered lentivirus plus 8 μg/mL polybrene (Sigma-Aldrich, St. Louis, MO, USA) for 24 h and then treated with 5 μg/mL puromycin (InvivoGen, San Diego, CA, USA) for more than 7 days to obtain stably transfected cells. Sequences of siRNAs, shRNAs, sgRNAs and primers used in the plasmid construction are shown in Additional file 1: Table S1.

### Cell proliferation and colony formation assays

The cell proliferation assay was performed using a cell counting kit-8 (CCK8; MCE, Monmouth Junction, NJ, USA). First, cells were plated at a density of 1000 cells/well in 96-well plates containing 100 µL of culture medium. The OD450 was measured for 2 h after adding 10 µL of CCK8 solution. For the colony-formation assay, cells were seeded onto a 12-well plate at a density of 1000 cells/well. After one week of culture, cells were treated with methanol and stained with 0.1% crystal violet. The number of visible colonies was counted by ImageJ software (https://imagej.net/).

### Ethynyl deoxyuridine (EdU) incorporation assay

Cell proliferation was also examined with a 5-Ethynyl-2’-deoxyuridine incorporation assay using an EdU Apollo DNA in vitro kit (RIBOBIO, Guangzhou, China) following the manufacturer’s instructions. Briefly, 5000 cells were seeded into 96-well plates and incubated with 100 μL of 50 μM EdU per well for 2 h at 37 °C. Next, cells were fixed with 4% paraformaldehyde and fixation was stopped by the addition of glycine. Subsequently, cells were stained with 1X Apollo solution and 1X Hoechst33342 solution. Finally, the cells were visualized under the Zeiss fluorescence photomicroscope.

### Cell invasion and migration assay

Cell invasion and migration assays were performed using Transwell chambers (Corning Costar, Tewksbury, MA, USA). 3 × 10^4^ cells were seeded into the upper chamber of each insert with 300 μL serum-free medium and 500 μL medium with 10% FBS added into the lower chambers. After incubating at 37 °C for 18 h, the cells were fixed with methanol and stained with 0.1% crystal violet. The cells on the top side of the membrane were removed using cotton swabs and the cells that had invaded to the underside of the membrane were then counted and imaged under a microscope (Leica DM4000, Buffalo Grove, IL, USA).

### Western blotting analysis

The total protein of cells was lysed with RIPA buffer (Beyotime, Shanghai, China) containing 1 mM PMSF (Beyotime). The concentration of protein was quantified using the Pierce™ BCA Protein Assay Kit (Thermo Scientific, Irwindale, CA, USA). 30 μg of protein samples were separated by 10% SDS-PAGE and transferred to a PVDF membrane (Millipore, Burlington, MA, USA). The membrane was blocked in 5% non-fat milk for one hour at room temperature and incubated with diluted primary antibody at 4 °C overnight. Next, the membrane was incubated with horseradish peroxidase (HRP)-labeled secondary antibody for one hour at room temperature. The signal was visualized using ECL reagent and photographed by ChemiDoc Touch Imaging System (Bio-Rad, Hercules, CA, USA). Information of antibodies for FUBP1, cMyc, METTL3, Cyclin A2, B1 and D1, and GAPDH can be found in Additional file 1: Table S2.

### Cell cycle assay

For cell cycle analysis, 10^6^ cells were collected and fixed with 70% ethanol overnight at 4 °C. After washing with PBS, cells were resuspended in 500µL of PI/RNase Staining Buffer (BD biosciences, San Jose, CA, USA) and incubated for 15 min at room temperature whilst being protected from light. A Cytoflex S flow cytometer (Beckman, Brea, CA, USA) was used to analyze cell cycle distribution.

### In vivo xenograft model

For the in vivo tumorigenicity assay, female BALB/c nude mice (ages 4–5 weeks) were randomly divided into two groups (*n* = 6/group). Calu1 cells (4 × 10^6^) that had been stably transfected with sh-LCAT3 or scramble were implanted subcutaneously into the nude mice. Tumor growth was measured after one week, and tumor volumes were calculated with the following formula: Volume (cm^3^) = (length × width^2^)/2. After four weeks, the mice were euthanized, and the tumors were collected and weighed. For the in vivo tumor invasion assay, 1.2 × 10^6^ scramble or shLCAT3 cells were injected intravenously into the tail vein of nude mice (*n* = 6/group). 1.5 mg luciferin (Gold Biotech, St Louis, MO, USA) was administered once a week for 4 weeks, to monitor metastases using an IVIS@ Lumina II system (Caliper Life Sciences, Hopkinton, MA, USA). All experiments were performed in accordance with the Guide for the Care and Use of Laboratory Animals (NIH publication 80–23, revised 1996), with the approval of Zhejiang University, Hangzhou, China.

### RNA sequencing (RNA-seq) and data analysis

Total RNA was isolated from Calu1 cells transfected with LCAT3 siRNA (si-LCAT3 #2, or si-LCAT3 #4) or negative control (NC) siRNA (*n* = 3). mRNA libraries were prepared with 5 μg of total RNA using a TruSeq RNA Sample Preparation Kit from Illumina as we have described previously [14]. mRNA libraries were multiplexed and sequenced with an Illumina HiSeq X10 sequencer (Additional file 1: Table S3). RNA-seq data were aligned to the human genome (hg19) using TopHat2 (v 2.0.13) [15]. Transcripts were assembled from RNA-seq alignments using Stringtie2 (v2.1.0) [16]. Expression was quantified by fragments per kilobase of transcript per million reads mapped (FPKM). Differentially expressed genes between LCAT3 knockdown and NC control cells were detected using Cuffdiff2 (v2.2.1) [17]. The false discovery rate (FDR) < 0.05 was used as a cutoff for claiming significant differential expression.

### Dot blotting assay

Tatal RNA was isolated using Trizol reagent. RNA was diluted to a certain concentration and denatured by heating 95 ℃ for 5 min. 2ul of RNA samples were loaded on an amersham hybond-n + membrane (GE Healthcare, Chicago, IL, USA). After UV crosslinking, the membrane was blocked with 5% non-fat milk for 1 h at room temperature, incubated with anti-m6A antibody (1:500, Milipore, Burlington, MA, USA) overnight at 4 °C and subsequently incubated with secondary antibody for 1 h at room temperature. After washed by PBST, signals were detected using a chemiluminescence system (Bio-Rad). The membrane was stained with 0.02% methylene blue in 0.3 mol/L sodium acetate (pH 5.2) as a loading control.

### MeRIP-seq and MeRIP-qPCR

mRNA was purified using a polyA Spin™ mRNA Isolation Kit (NEB, Ipswich, MA USA). Methylated RNA immunoprecipitation (MeRIP) was performed according to the instructions of the Magna MeRIP™ m6A Kit (Merck, Darmstadt, Germany). Briefly, 18 μg of mRNA was fragmented into approximately 100-nt oligonucleotides using a fragmentation buffer. Then the post-fragmentation size distribution was validated using an Agilent 2100 Bioanalyzer with an Agilent RNA 6000 Kit. The Magna ChIP Protein A/G Magnetic Beads were incubated for 1 h at room temperature with m6A-specific antibody in immunoprecipitation buffer. The mixture was then incubated with the MeRIP reaction mixture overnight at 4 °C. Eluted RNA was then prepared for RNA-seq libraries as described above (Additional file 1: Table S4) or for qRT-PCR analysis. Peak calling was conducted using R package exomePeak2 (v1.2.0) with default parameter settings [18], and target peaks were visualized by R package wiggleplotr (v1.14.0) (https://bioconductor.org/packages/release/bioc/html/wiggleplotr.html).

### RNA pull-down assay

Biotin-labeled targeted LCAT3 probes #1-#8, antisense probes #1-#4 and lacZ probes (negative control) #1-#2 were synthesized by Life Technologies (Carlsbad, CA, USA). The RNA pull-down assay was performed using a Pierce™ Magnetic RNA-Protein Pull-Down Kit (Thermo scientific, Rockford, IL, USA) according to the manufacturer’s instructions. Briefly, 100 pmol biotin labeled anti-sense RNA probes, sense RNA probes and negative control probes were incubated with streptavidin beads for one hour at room temperature. Subsequently, equal amounts of lysate derived from Calu1 cells were incubated with probe-beads complex overnight at 4 °C. After the pull down, the proteins were eluted by 1 × SDS loading buffer. The eluate was then separated by 4–12% sodium dodecyl sulfate polyacrylamide gel electrophoresis and stained with silver staining.

### MS2-tagged RNA affinity purification

pcDNA3.1-MS2 plasmid was a gift of Professor Huai Qiang Ju (Sun Yat-sen University Cancer Center, Guangzhou, China). MCP-3xFLAG plasmid was obtained from OBiO Technology (Shanghai, China). Calu1 cells were cotransfected with pcDNA3.1-LCAT3-MS2 and MCP-3xFLAG plasmids. After 48 h, the cells were cross-linked with 37% formaldehyde for 10 min at room temperature, followed by 1.25 M glycine quenching for 5 min. Then, the cells were lysed on ice for 10 min and centrifuged at 14,000×*g* for 10 min. The proteins were immunoprecipitated with an anti-FLAG antibody (Sigma-Aldrich). The RNA/protein immunoprecipitations were boiled in loading buffer, and proteins were detected by western blotting analysis.

### RNA immunoprecipitation (RIP)

The RIP assay was performed using an EZ-Magna RIP Kit (Millipore, Bedford, MA, USA) according to the manufacturer’s instructions. Briefly, approximately 2 × 10^7^ cells were lysed with 115 μL RIP Lysis Buffer for per immunoprecipitation. 5 µg of the antibody of interest was incubated with Protein A/G Magnetic Beads for 30 min at room temperature. 100 µL of RIP lysate was added to each bead-antibody complex in a RIP Immunoprecipitation Buffer and incubated overnight at 4 °C. Each immunoprecipitant was resuspended in proteinase K buffer and incubated at 55 °C for 30 min. RNA was isolated by phenol, chloroform and isoamyl alcohol according to the manufacturer’s instructions, and was detected by qRT-PCR.

### RNA stability assay

A549 and Calu1 cells were treated with 5 µg/mL actinomycin D (Sigma-Aldrich) to inhibit transcription. Cells were then collected at various time intervals. LCAT3 and MYC mRNA levels were assessed using qRT-PCR.

### Protein stability assay

A549 and Calu1 cells were treated with 100 µg/mL cycloheximide (CHX) for the indicated times and harvested. Protein expression of c-Myc was then determined by western blot analysis.

### Luciferase reporter assays

The MYC promoter containing the far-upstream element (FUSE) sequence or lacking the FUSE sequence was sub-cloned and inserted into the pGL3-basic vector (Promega, Madison, WI, USA). 293T cells were seeded onto 24-well plate and transfected with LCAT3 or FUBP1 siRNA. After incubation for 48 h, the cells were lysed in 1 × Passive lysis. A Dual-Luciferase Reporter Assay System (Promega) was applied to measure the firefly luciferase activity, with Renilla luciferase serving as a transfection control.

### Chromatin immunoprecipitation (ChIP)

Briefly, approximately 5 × 10^6^ cells were fixed with 1% formaldehyde (Sigma-Aldrich) for 10 min at room temperature. Fixation was stopped by the addition of glycine and further incubation was conducted for 5 min. Chromatin was digested to 150–900 bp using micrococcal nuclease and a sonicator (for 4 min alternating 20 s on and 20 s off). 10–20 µg of digested, cross-linked chromatin was used per immunoprecipitation. The antibody–chromatin complex was captured with Pierce Protein A/G Magnetic Beads. ChIP DNA was extracted and analyzed by qPCR.

### Statistical analysis

Data are presented as mean ± standard deviation of three independent experiments. Two-sided Student’s t test was used to analyze the differences between groups. KEGG pathways that were enriched for differentially expressed genes (DEGs) were detected using hypergeometric tests. Gene Ontology (GO) terms that were enriched for DEGs were evaluated using Fisher's exact tests. Kaplan–Meier curves were plotted to reveal survival differences between two groups of patients according to their expression levels. A log-rank test was used to evaluate statistical differences in survivals. Statistical significance was assigned at *P* < 0.05. All statistical analyses were implemented in the statistical package R (https://www.r-project.org/).

## Results

### LCAT3 is upregulated in lung cancer, which is positively associated with poor prognosis

To characterize novel lncRNAs deregulated in lung cancer, we analyzed the expression profiles in 485 LUAD tissues and 56 adjacent normal tissues from The Cancer Genome Atlas (TCGA). We identified 93 lncRNAs that were differentially expressed between lung tumors and adjacent normal tissues, and were also associated with prognosis in LUAD patients [14]. Our previous study demonstrated that a lncRNA, designated as LCAT1, functions as a ceRNA to regulate the expression and function of RAC1 through competitively binding with miR-4715-5p [14]. In the present study, we focused on another novel lncRNA, designated as LCAT3 with unknown function in lung cancer.

**Fig. 1:**
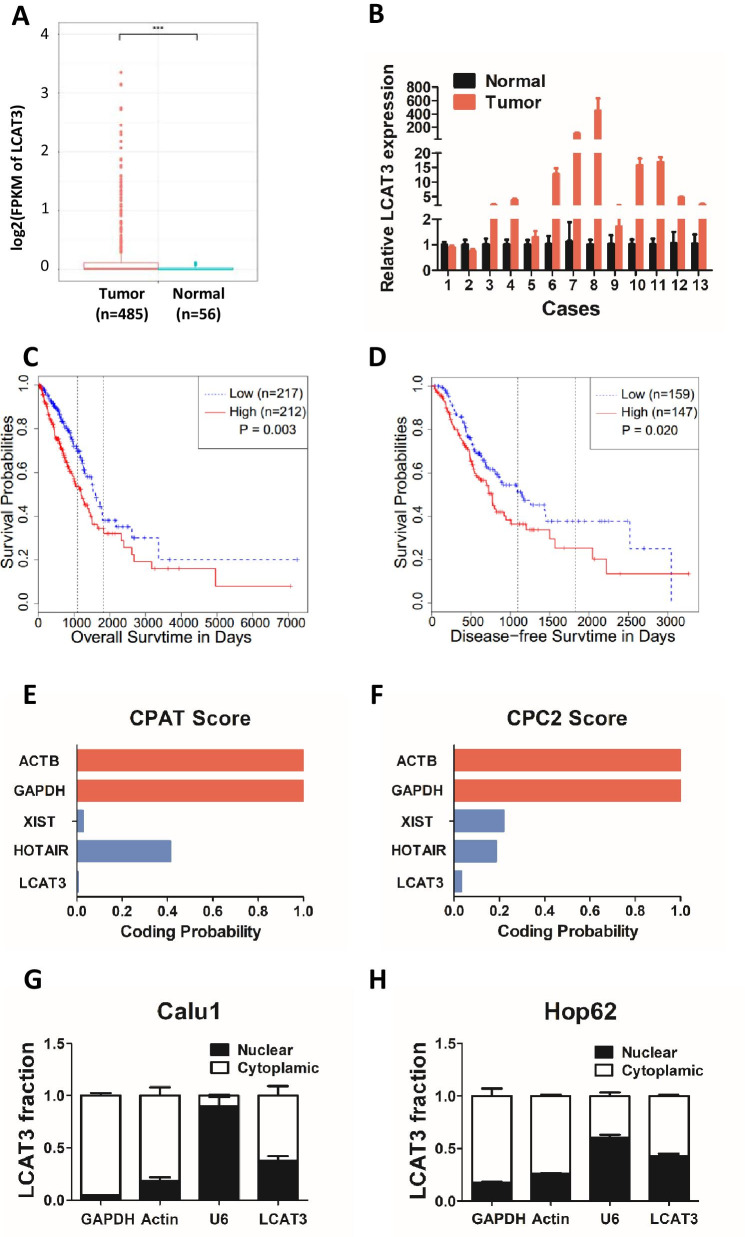
LCAT3 is upregulated in lung tumor tissues and associated with poor prognosis of LUAD. **A** Boxplot showing the relative expression of LCAT3 in lung tumors (*n* = 485) and adjacent normal (*n* = 56) tissues in a TCGA LUAD cohort. LCAT3 expression was quantified by FPKM in the RNA-seq data. **B** LCAT3 expression validated by qRT-PCR in an independent cohort of 13 paired samples of LUAD tissues and adjacent normal tissues. LCAT3 expression was normalized to the expression of β-actin. **C**, **D** Kaplan–Meier curves of overall survival (**C**) and disease-free survival (**D**) of lung cancer patients with high *versus* low expressions of LCAT3. **E**, **F** The protein coding potential of LCAT3 evaluated by the Coding Potential Assessment Tool (CPAT) and Coding Potential Calculator 2 (CPC2). Two verified lncRNAs, XIST and HORAIR, served as controls for the prediction. **G**, **H** Subcellular localization of LCAT3. Real-time PCR analysis confirmed the nuclear and cytoplasmic fraction of LCAT3 transcript in Calu1 and Hop62 cells; U6 and GAPDH served as positive controls for the nuclear and cytoplasmic fractions, respectively

LCAT3 is located on human chromosome 3q13.31 and has one transcript with five exons. The 5′ and 3′ cDNA RACE assays revealed that LCAT3 is a long non-coding RNA of 1541 bp in length (Additional file 1: Fig. S1). Analysis of RNA-seq data from TCGA indicated that LCAT3 was remarkably upregulated in LUAD tissues compared with adjacent normal tissues (Fig. 1A). We further verified the expression of LCAT3 in 13 pairs of lung cancer samples and corresponding noncancerous lung samples using qRT-PCR. Consistent with the RNA-seq data, the qRT-PCR results confirmed that LCAT3 was upregulated in lung cancer tissues (Fig. 1B). Importantly, the Kaplan–Meier survival analysis revealed that LUAD patients with higher LCAT3 expression level had shorter overall survival and disease-free survival than those with lower LCAT3 expression level (Fig. 1C, D). Furthermore, the Coding Potential Assessment Tool (CPAT) and Coding Potential Calculator 2 (CPC2) were used to calculate the protein-coding capacity of LCAT3. As expected, the LCAT3 had a lower potential to encode protein than other classic lncRNAs such as XIST and HOTAIR (Fig. 1E, F), indicating LCAT3 is most likely a lncRNA. Subcellular localization assays demonstrated that LCAT3 is distributed in both the nucleus and cytoplasm (Fig. 1G, H).

### METTL3 is responsible for the upregulation of LCAT3 in lung cancer

We next explored the underlying mechanism by which LCAT3 is up-regulated in LUAD. We analyzed the copy number variation (CNV) data of LUAD in TCGA as previously described [14]. There was no significant association between LCAT3 CNV and its expression (Additional file 1: Fig. S2A, B). We then checked potential regulation elements (such as transcription factor binding and histone modification) in LCAT3 using UCSC Genome Browser, but there were no such regulation elements near the LCAT3 locus (Additional file 1: Fig. S2C). Finally, we focused on the effects of epigenetic modification on LCAT3 expression, including DNA methylation and RNA methylation. 5-Aza-2ʹ-Deoxycytidine (5-Aza-dC) acts as a DNA methyltransferase inhibitor. When lung cancer cells were treated with 5-Aza-dC, the expression of LCAT3 was not sharply altered (Additional file 1: Fig. S2D–F).

**Fig. 2:**
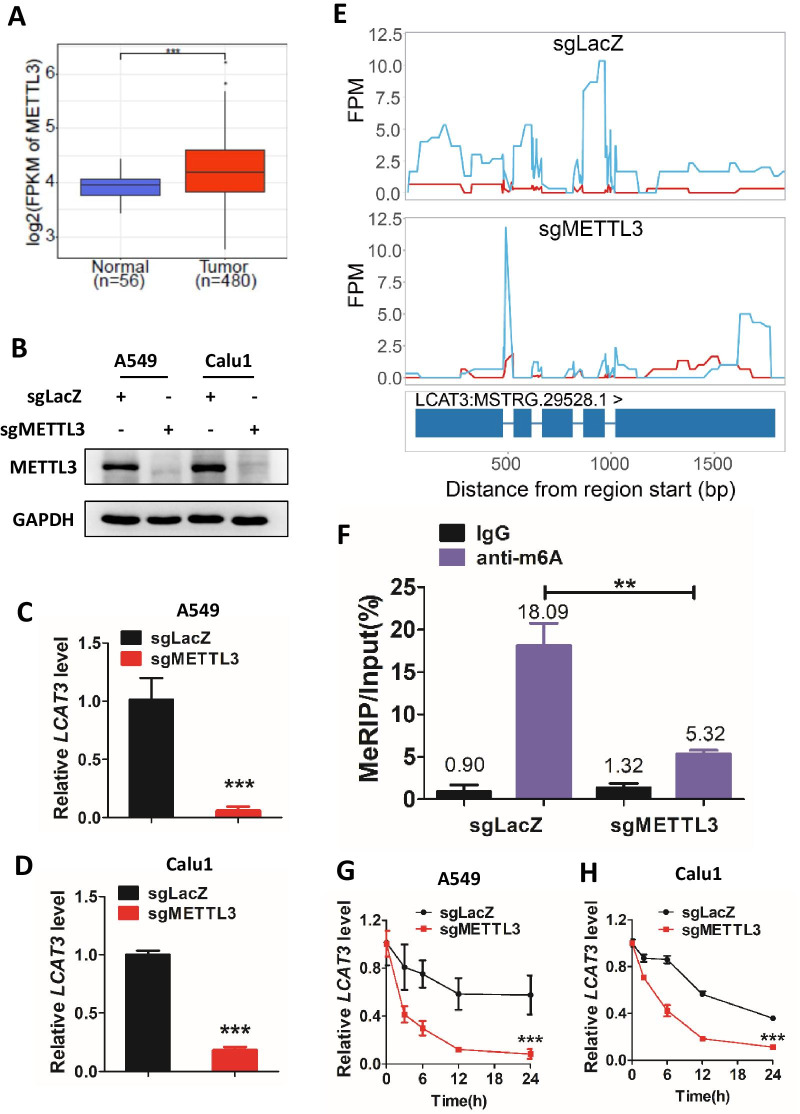
LCAT3 is upregulated by METTL3 through m6A modification. **A** METTL3 is upregulated in LUAD. Boxplot showing the relative expression of METTL3 in lung tumors (*n* = 480) and adjacent normal tissues (*n* = 56) in TCGA LUAD cohort. METTL3 expression was quantified by FPKM in the RNA-seq data. **B** Western blot assays to detect METTL3 knockout efficiency in A549 and Calu1 cells. **C**, **D** qRT-PCR analysis of LCAT3 expression in sg-lacZ and sg-METTL3 lung cancer cells. **E** MeRIP-seq of sgLacZ (top) and sgMETTL3 (bottom) A549 cells showed that depletion of METTL3 reduces the m6A modification on LCAT3. Red line represents input group while blue line represents IP group. **F** MeRIP-RT-qPCR confirmed the change in m6A levels in METTL3-knockout A549 cells. **G**, **H** Half-life of LCAT3 in sg-lacZ and sg-METTL3 lung cancer cells treated with 5 μg/mL Actinomycin D

On the other hand, m6A modification is the most prevalent posttranscriptional modifications of mRNA and lncRNA, and regulates it translation, stability, etc. [19, 20]. Our bioinformatics analysis revealed that METTL3, the core m6A methyltransferase, was indeed upregulated in LUAD (Fig. 2A). We thus knocked out METTL3 using the CRISPR/Cas9 system in lung cancer cells, and the knockout efficiency was confirmed by both western bolt (Fig. 2B) and dot blot (Additional file 1: Fig. S2G). We found that depletion of METTL3 significantly reduced LCAT3 expression levels (Fig. 2C, D). Furthermore, the MeRIP-seq indicated an enrichment of m6A modification on LCAT3, and a reduction in m6A levels upon METTL3 knockout in A549 cells (Fig. 2E). Subsequent MeRIP-qPCR confirmed that METTL3 knockout remarkably reduced LCAT3 m6A modification levels (Fig. 2F). Finally, we treated lung cancer cells with actinomycin D to block transcription and found that METTL3 knockdown significantly decreased the half-life of LCTA3 transcript (Fig. 2G, H). Taken together, METTL3-mediated m6A appears to be responsible for the upregulation of LCAT3 in LUAD, likely by stabilizing its transcript.

### LCAT3 promotes proliferation of lung cancer cells both in vitro and in vivo

**Fig. 3:**
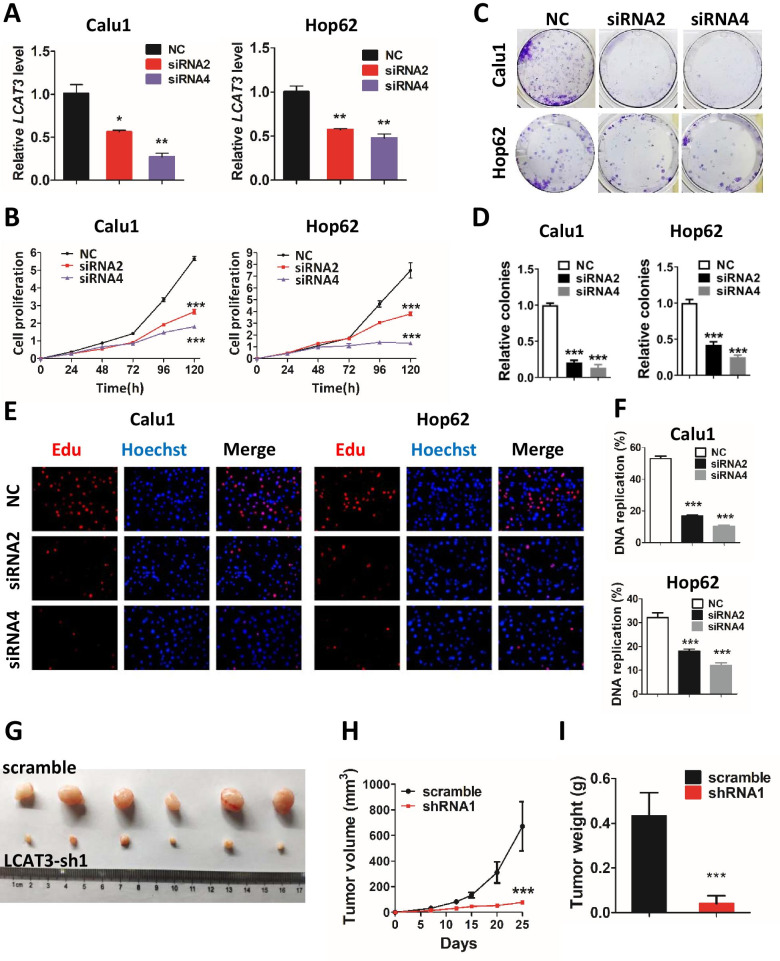
LCAT3 promotes proliferation of lung cancer cells both in vitro and in vivo. **A** qRT-PCR analysis of LCAT3 expression in Calu1 and Hop62 cells transfected with LCAT3 siRNA (siRNA2 or siRNA4) and negative control (NC) siRNA. **B** CCK-8 assays were performed to assess cell proliferation in NC siRNA and LCAT3 knockdown lung cancer cell lines. **C** Colony formation assays were performed to assess colony formation abilities in NC siRNA and LCAT3 knockdown lung cancer cell lines. **D** The number of colonies were counted from three independent assays. **E** EdU assays of Calu1 and Hop62 cells transfected with NC siRNA and LCAT3 siRNA. **F** The DNA replication ratio was determined from three independent assays. **G** Representative images of the xenograft tumors in mice from LCAT3 knockdown and control groups. Calu1 cells stably transfected with shLCAT3 or scramble control were subcutaneously inoculated into the flanks of mice. **H**, **I** Quantification of tumor volume (**H**) and tumor weight (**I**) in xenograft mouse models from LCAT3 knockdown and control groups

To investigate the biological function of LCAT3 in lung cancer cells, we silenced LCAT3 in Calu1 and Hop62 lung cancer cells using siRNAs, which sufficiently reduced the expression of LCAT3 (Fig. 3A). CCK-8 assays revealed that cell proliferation was significantly suppressed after the silencing of LCAT3 (Fig. 3B). Similarly, the colony formation assays have also shown that LCAT3 knockdown could significantly reduce cell colonies compared with siRNA-NC (Fig. 3C, D). Moreover, EdU assays showed that LCAT3 depletion significantly decreased DNA replication in lung cancer cells (Fig. 3E, F). Consistently, LCAT3 overexpression in A549 and Calu1 cells (Additional file 1: Fig. S3A) promoted proliferation (growth) and survival (colony formation) of lung cancer cells (Additional file 1: Fig. S3B, C).

To further evaluate the oncogenic role of LCAT3 in vivo, we stably knocked down LCAT3 in Calu1 and Hop62 cells with shRNA (Additional file 1: Fig. S4A) and established xenograft mouse models by subcutaneous injection (*n* = 6 per group) (Fig. 3G). CCK-8 assays revealed that cell proliferation was significantly suppressed after LCAT3 knockdown (Additional file 1: Fig. S4B). The tumor volume and tumor weight were significantly decreased in the LCAT3 knockdown group, as compared with the shRNA NC group (Fig. 3H, I). Collectively, LCAT3 appears to be essential for the growth and survival of lung cancer cells both in vitro and in vivo.

### Effects of LCAT3 on migration, invasion, and cell cycle progression of lung cancer cells

**Fig. 4:**
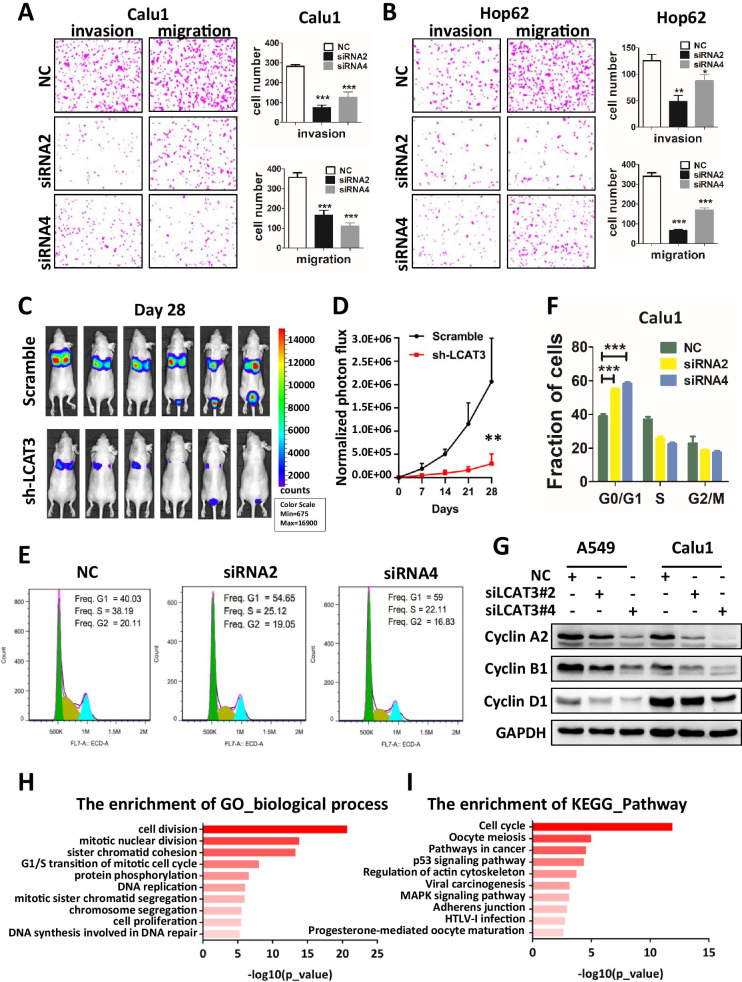
Effects of LCAT3 on migration, invasion, and cell cycle of lung cancer cells. **A**, **B** Transwell invasion and migration assays in Calu1 (**A**) and Hop62 (**B**) cells. Representative images and statistical analysis of three independent assays are shown. **C** LCAT3 promotes lung cancer cell metastasis in vivo. 1.2 × 10^6^ scramble or shLCAT3 cells were injected intravenously into the tail vein of nude mice (*n* = 6/group). The metastatic lung colonization in nude mice was measured by bioluminescence imaging. **D** Quantification of photon flux for metastases by tail-vein injection of shLCAT3 or scramble control Calu1 cells in nude mice. **E** Cell cycle analysis by flow cytometry was performed to determine the percentage of cells in different cell cycle phases. **F** Bar chart showing the percentage of cells in G1–G0, S, or G2–M phase. **G** Western blot analysis of cyclin A2, cyclin B1 and cyclin D1 in Calu1 and Hop62 cells transfected with LCAT3 siRNA or NC siRNA. **H** Gene ontology (GO) analysis was conducted to identify biological processes enriched for differentially expressed genes in Calu1 cells upon knockdown of LCAT3. **I** KEGG pathway analysis was conducted to identify pathways enriched for differentially expressed genes in Calu1 cells upon knockdown of LCAT3

Next, we explored the effects of LCAT3 on migration and invasion of lung cancer cells. Transwell assays revealed that LCAT3 knockdown inhibited the migration and invasion of Calu1 and Hop62 cells (Fig. 4A, B, Additional file 1: Fig. S4C, D). To explore whether LCAT3 also regulate lung cancer metastasis in vivo, luciferase-labelled control or LCAT3-silencing Calu1 cells were injected intravenously into nude mice which were then subjected to bioluminescent imaging (BLI) once a week. Continued BLI monitoring revealed a dramatic decrease of metastatic outgrowth in the lungs of mice injected with LCAT3-silencing Calu1 cells over 4 weeks (Fig. 4C, D).

To determine whether LCAT3 knockdown had affected the cell cycle in lung cancer cells, flow cytometry assays were performed in LCAT3 knockdown and NC lung cancer cells. LCAT3 knockdown caused the G1 arrest (Fig. 4E, F). Western blot assays confirmed that several cell cycle-related proteins such as cyclin A2, cyclin B1 and cyclin D1 were obviously downregulated upon LCAT3 knockdown (Fig. 4G).

To further analyze the effect of LCAT3 on lung cancer, RNA-seq analysis was performed to compare the gene expression profiles of LCAT3 siRNA and control siRNA. A total of 1221 genes were identified as significantly altered expressed in LCAT3-silenced Calu1 cells compared with the control cells (FDR < 0.05) (Additional file 1: Fig. S5A). These significantly altered genes were then subjected to the enrichment analysis of KEGG pathways and Gene ontology (GO) terms. Interestingly, the most significant KEGG pathway and GO term are related cell cycle and cell division, indicating that LCAT3 silencing resulted in significant alterations of cell cycle regulatory genes (Fig. 4H, I). Several representative cell cycle related genes were selected for further validation using qRT-PCR, and consistent results were obtained with RNA-seq analysis (Additional file 1: Fig. S5B, C).

### LCAT3 physically interacts with FUBP1, which is also overexpressed in LUAD

**Fig. 5:**
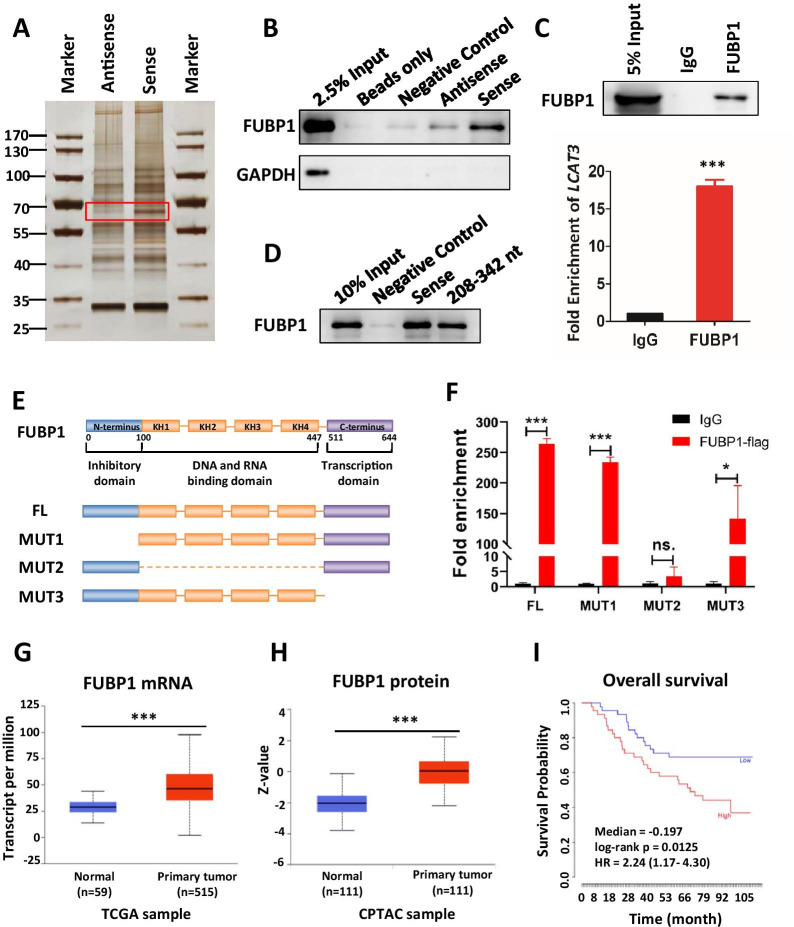
LCAT3 physically interacts with FUBP1. **A** Proteins interacting with LCAT3 were identified by pull down assay followed by mass spectrometry analysis. **B** Western blot analysis of FUBP1 in beads only, negative control, sense and antisense LCAT3 pull-down fractions. GAPDH served as a negative control. **C**. FUBP1 RIP assays in Calu1 cells. A western blot assay confirmed FUBP1 immunoprecipitation (Up); The relative fold enrichment of LCAT3 between FUBP1 and IgG RIP fractions was determined by qRT-PCR (Down). **D** LCAT3 sense and 208–342 nt fragments were performed with RNA pull down and western blotting assays. **E** The schematic structure of full-length FUBP1 proteins (FL: 1–644) and three domain-deleted mutants (MUT1: 100–644; MUT2: 1–100 and 447–644; MUT3: 1–511) of FUBP1 variants used in this study. The blue box is the inhibitory domain, the orange box is the DNA and RNA binding domain, and the purple box is the transcription domain. **F** RIP assays were performed using anti-FLAG antibodies in Calu1 cells that were transfected with vectors expressing the FLAG-tagged FL and the deleted mutants (MUT1-3) of FUBP1. **G** Relative mRNA expression of FUBP1 primary tumors (*n* = 515) and in adjacent normal tissues (*n* = 59) from TCGA LUAD samples. **H** Relative protein expression of FUBP1 in primary tumors (*n* = 111) and adjacent normal tissue (*n* = 111) from Clinical Proteomic Tumor Analysis Consortium (CPTAC) LUAD samples. **I** Kaplan–Meier survival analysis of overall survival of lung cancer patients using FUBP1 expression. The data were downloaded from the study of Takeuchi et al. (GEO accession: GSE11969; No. patients: 90)

To elucidate the potential molecular mechanisms of LCAT3 action in lung cancer cells, we performed RNA pull-down assays to identify LCAT3 binding proteins. Briefly, the retrieved proteins from pull-down fractions were subjected to SDS-PAGE electrophoresis and silver staining. The distinct band between LCAT3 sense and antisense, which was weighted between 60 and 70 kDa, was cut from the gel for mass spectrometry (MS) analysis (Fig. 5A). Proteins of the correct molecular weight were selected, and nonspecific binding proteins on antisense were excluded (Additional file 1: Table S5). FUBP1 was chosen for further study owning to its specific binding with LCAT3 (Fig. 5B, Additional file 1: Fig. S6A). In addition, RIP assays were performed with an anti-FUBP1 antibody to further demonstrate the interaction between FUBP1 and LCAT3 (Fig. 5C).

A series of binding region assays were then performed to explore the binding sites between LCAT3 and FUBP1. Briefly, the secondary structure of LCAT3 was analyzed with RNAfold (Additional file 1: Fig. S6B) [21]. The interaction profile of LCAT3 and FUBP1 was predicted by catRAPID (Additional file 1: Fig. S6C) [22]. These bioinformatics analyses showed that 208–342 nt of LCAT3, which formed a stem-loop structure, was the core sequence binding to FUBP1. Therefore, we used the 208–342 nt fragments of LCAT3 for RNA pull-down assays. The results confirmed that this stem-loop region (208–342 nt) was indeed responsible for the binding between LCAT3 and FUBP1 (Fig. 5D). The same result was obtained using MS2-tagged RNA affinity purification (MTRAP) (Additional file 1: Fig. S6D). Previous studies have shown that FUBP1 can be divided into three domains, which consist of inhibitory domain, DNA and RNA binding domain and transcription domain [23]. Therefore, we constructed Flag-tagged full-length wild type and three domain-deleted mutants of FUBP1 variants (Fig. 5E). The RIP assay showed that LCAT3 mainly bound to the DNA and RNA binding domain (100-447aa) (Fig. 5F). Taken together, these data suggest that LCAT3 physically interacts with FUBP1, and the stem-loop region (208–342 nt) of LCAT3 and the DNA and RNA binding domain (100-447aa) of FUBP1 were essential for the binding between LCAT3 and FUBP1.

FUBP1 is a master regulator of transcription, splicing, and translation through its bindings to single-stranded DNA and RNA [23–26]. In particular, the overexpression of FUBP1 has been observed in a growing number of cancers, leading to a deregulation of various targets, including the fine-tuned MYC oncogene [27–30]. We found that both mRNA and protein levels of FUBP1 were significantly upregulated in LUAD (Fig. 5G, H), and the high expression of FUBP1 was associated with shorter survival time in lung cancer patients (Fig. 5I).

### FUBP1 silencing inhibits proliferation and survival of lung cancer cells

**Fig. 6:**
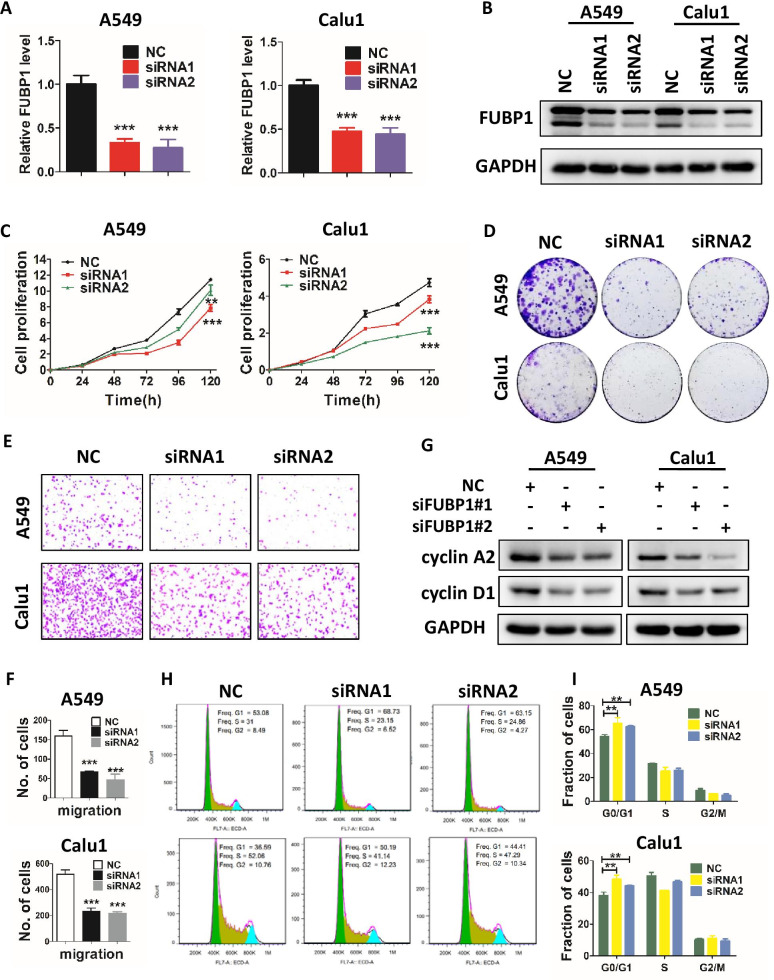
FUBP1 knockdown inhibits proliferation, migration, and the cell cycle of lung cancer cells. **A** qRT-PCR analysis of FUBP1 expression in lung cancer cells transfected with FUBP1 siRNA (siRNA1 or siRNA2) or NC siRNA. **B** Western blot analysis of FUBP1 expression in lung cancer cells transfected with FUBP1 siRNA or NC siRNA. **C** CCK-8 assays for A549 and Calu1 cells transfected with FUBP1 siRNA or NC siRNA. **D** Colony formation assays for A549 and Calu1 cells transfected with FUBP1 siRNA or NC siRNA. **E**, **F** Transwell migration assays for FUBP1-silenced and control lung cancer cells. Representative images (**E**) and statistical analysis of three independent assays (**F**) are shown. **G** Western blot analysis of cyclin A2 and cyclin D1 in A549 and Calu1 cells transfected with FUBP1 siRNA or negative control siRNA. **H**, **I** Cell cycle analysis by flow cytometry was performed to determine the percentage of cells in different cell cycle phases (**H**). Bar chart showing the percentage of cells in G1–G0, S, or G2–M phase (**I**)

The biological functions of FUBP1 in LUAD have not yet been reported, we, therefore, knocked down FUBP1 in A549 and Calu1 cells by siRNAs to assess potential changes in tumor cell phenotypes. Upon FUBP1 knockdown (Fig. 6A, B), we observed a significant inhibition of growth (Fig. 6C) and colony survival (Fig. 6D) of lung cancer cells. Furthermore, like LCAT3 knockdown, FUBP1 knockdown also suppressed the migration of A549 and Calu1 lung cancer cells (Fig. 6E, F), and triggered G1 arrest (Fig. 6H, I) by significantly reducing the levels of cyclin A2 and cyclin D1 (Fig. 6G). Collectively, FUBP1 also plays an oncogenic role by enhancing malignant phenotypes of lung cancer cells.

### LCAT3 and FUBP1 co-regulate the expression of c-Myc

**Fig. 7:**
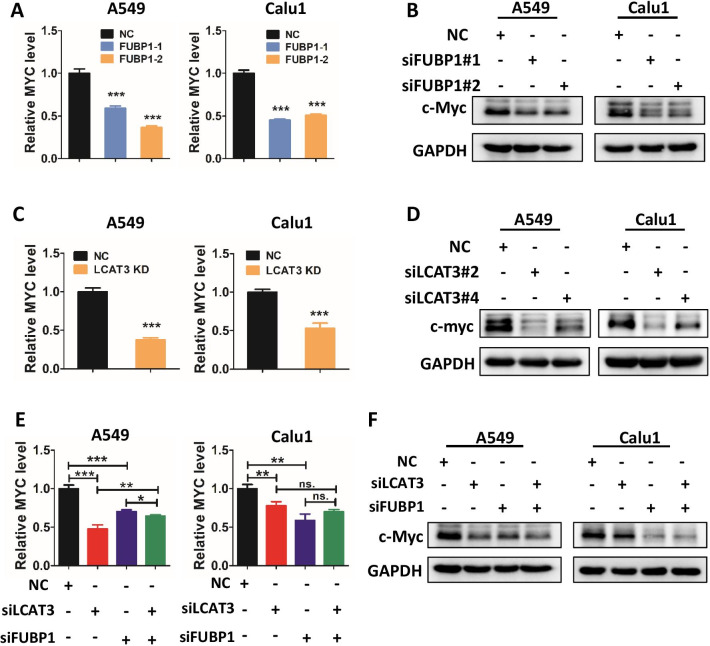
LCAT3 and FUBP1 co-regulate the expression of c-Myc. **A**, **C** qRT-PCR detecting the mRNA expression of MYC in A549 and Calu1 cells with FUBP1 knockdown (**A**) and LCAT3 knockdown (**C**), respectively. **B**, **D** Western blot assays detecting the protein expression of c-Myc in A549 and Calu1 cells with FUBP1 knockdown (**B**) or LCAT3 knockdown (**D**), respectively. **E** qRT-PCR detecting the mRNA expression of MYC in A549 and Calu1 cells transfected with LCAT3 or FUBP1 siRNA. **F** Western blot assays detecting the protein expression of c-Myc in A549 and Calu1 cells transfected with LCAT3 and/or FUBP1 siRNA or NC siRNA

To investigate whether a cross-talk exists between LCAT3 and FUBP1, we first assessed FUBP1 levels in LCAT3-silenced lung cancer cells and found no change (Additional file 1: Fig. S7A, B). Given that FUBP1 is known to upregulate MYC transcription by binding to the FUSE sequence [31], and our data showed LCAT3 regulates cell cycle progression, we, therefore, tested our working hypothesis that LCAT3 would collaborate with FUBP1 to regulate MYC expression. Like FUBP1 knockdown, which suppressed the levels of c-MYC mRNA and protein as expected (Fig. 7A, B), LCAT3 knockdown also significantly reduced the levels of c-MYC mRNA and protein in A549 and Calu1 cells (Fig. 7C, D). Consistently, over-expression of either LCAT3 or FUBP1 up-regulated the MYC levels (Additional file 1: Fig. S7C–S7F). Thus, like FUBP1, LCAT3 is also a c-MYC positive regulator. Interestingly, the c-MYC levels, reduced by knockdown of either LCAT3 or FUBP1, cannot be further reduced by simultaneous knockdown of both LCAT3 and FUBP1 (Fig. 7E, F), suggesting that LCAT3 and FUBP1 act at the same regulatory point to control c-MYC expression.

### LCAT3 recruits FUBP1 to activate MYC transcription

**Fig. 8:**
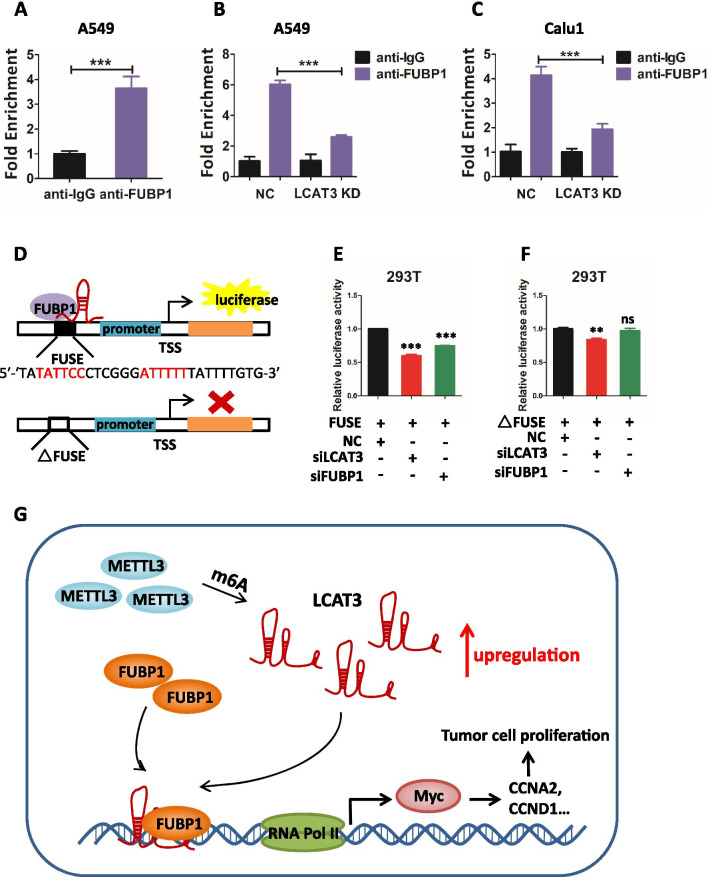
LCAT3 recruits FUBP1 to activate MYC expression. **A** qRT-PCR showing the interaction of FUBP1 with the MYC FUSE sequence in A549 cells from the ChIP assay. IgG was used as negative control. **B**, **C** ChIP analysis showing that the depletion of LCAT3 decreases the enrichment of FUBP1 at MYC FUSE sequence both in A549 (**B**) and Calu1 cells (**C**). **D** A schematic diagram of luciferase report assay. **E** MYC FUSE and promoter sequences (+ 33 to − 1896 bp) were constructed into pGL3 plasmid and subjected to luciferase reporter assays in either LCAT3 or FUBP1-silenced 293T cells. **F** FUSE-deficient MYC promoter sequences (+ 33 to − 1647 bp) were constructed into pGL3 plasmid and subjected to luciferase reporter assays in either LCAT3 or FUBP1-silenced 293T cells. **G** Proposed model for the LCAT3-FUBP1-MYC axis regulating the proliferation, migration, and invasion of lung cancer cells

To investigate how LCAT3 and FUBP1 cross-talk with each other to regulate MYC expression, we examined the stability of c-MYC mRNA and protein in A549 and Calu1 cells upon knockdown of either LCAT3 or FUBP1. Surprisingly, the LCAT3 knockdown had no effect on the half-life of c-MYC mRNA (Additional file 1: Fig. S8A, S8C), or c-MYC protein (Additional file 1: Fig. S8B, S8D). Consistently, FUBP1 knockdown had very little effect on the stability of c-MYC mRNA and protein (Additional file 1: Fig. S8E–S8H). We, therefore, hypothesized that LCAT3 and FUBP1 may work together to regulate MYC expression at the transcriptional level. Indeed, the ChIP assay showed that FUBP1 bound to the c-MYC promoter in lung cancer cells (Fig. 8A, Additional file 1: Fig. S9A), which is significantly reduced upon LCAT3 knockdown (Fig. 8B, C, Additional file 1: Fig. S9B, C). Thus, LCAT3 appears to be required for FUBP1 binding to c-MYC promoter. We then tested whether LCAT3 activates c-MYC expression by recruiting FUBP1 to the FUSE sequence on c-MYC promoter. Using luciferase reporter driven by c-MYC promoter in the presence or absence of FUSE sequence (Fig. 8D), we found that the c- MYC promoter activity was suppressed upon knockdown of either LCAT3 or FUBP1 in a manner dependent of FUSE sequence (Fig. 8E, F). Collectively, it appears that LCAT3 recruits FUBP1 to the MYC FUSE sequence to activate its transcription.

Finally, we analyzed the effect of LCAT3 and FUBP1 on several cell cycle regulatory genes, known to be MYC targets in lung cancer cells. The qPCR assays revealed that knockdown of either LCAT3 or FUBP1 significantly decreased the expression of E2F2, cyclinA2, cyclinB1, cyclinD1, CDK1 and CDK6 (Additional file 1: Fig. S5B, C, S9D, E), indicating that c-MYC transcription is indeed regulated by both LCAT3 and FUBP1.

## Discussion

Approximately 2% of the human genome encodes for proteins, while most are transcribed into non-coding RNAs [32, 33]. LncRNAs comprise the largest proportion of noncoding transcripts. In the past few years, thousands of lncRNAs have been annotated, but few have been functionally characterized [34]. In the present study, we identified a novel lncRNA, LCAT3, using LUAD RNA-seq data from TCGA and characterized it experimentally. LCAT3 displays a remarkable trend of increased expression in LUAD tissues compared with adjacent normal tissues. Importantly, higher LCAT3 expression levels predict poor prognosis in lung cancer patients. Furthermore, LCAT3 showed strong oncogenic activity by promoting proliferation, survival, migration and invasion of lung cancer cells. The in vivo xenograft model confirmed that LCAT3 knockdown inhibited tumor growth and metastasis in nude mice.

The m6A is the most prevalent internal RNA modification in eukaryotes, which is installed by methyltransferases (writers), erased by demethylases (erasers), and recognized via m6A-sepecific binding proteins (readers) [35, 36]. METTL3 is the key component of the m6A methyltransferase complex and has recently been reported to be responsible for lncRNA stability in tumor progression [37–39]. In recent years, a growing number of studies have shown that m6A functions as a critical post-transcriptional modification that regulates the biogenesis of both mRNAs and non-coding RNAs [35, 40]. In this study, we found that METTL3 is up-regulated in lung cancer, and overexpressed METTL3 mediates m6A modification of LCAT, leading to its stabilization, thus providing molecular insight for LCAT3 overexpression in LUAD.

LncRNAs typically exert their biological functions by physically interacting with regulatory proteins or acting as a miRNA sponge. For instance, glycolysis-associated lncRNA of colorectal cancer (GLCC1) stabilizes c-Myc from ubiquitination degradation by direct interaction with the HSP90 (HSP90AA1) chaperon, thereby reprograming the glycolytic metabolism for colorectal cancer [41]. On the other hand, lncRNA urothelial cancer associated 1 (UCA1) positively regulates the expression of Delta-like ligand 4 (DLL4) through sponging miR-182-5p, and subsequently promotes malignant phenotypes of renal cancer [42]. Our RNA pull-down assay and MS analysis demonstrated that LCAT3 directly interacts with FUBP1 and subsequently up-regulates the expression of c-MYC. Significantly, we observed a 50% decrease in c-MYC mRNA and a more than 80% decrease in c-MYC protein after silencing of either LCAT3 or FUBP1, indicating a mechanism of LCAT3 action on c-MYC via FUBP1.

FUBP1 is a member of FUSE-binding proteins (FBPs) family, which recognize single-stranded DNA and RNA [23, 43]. Our study revealed that FUBP1 acts as an oncoprotein in lung cancer, which promotes the G1 to S phase (G1/S) transition and cell proliferation. Both c-MYC (MYC) and p21 (CDKN1A) have been identified as FBP downstream effectors [44–46]. Through the luciferase-based reporter assay and immunoprecipitation-based ChIP assay, we demonstrated that LCAT3 recruited FUBP1 to the c-MYC promoter on the FUSE sequence/motif, thereby activating c-MYC transcription. Given c-MYC, a typical oncogenic transcription factor, regulates at least 15% of genes involved in cell proliferation, differentiation and metabolism [47], targeting the LCAT3/FUBP1/c-MYC axis could be a promising strategy for LUAD treatment.

## Conclusions

In conclusion, we identified and characterized LCAT3, a novel m6A-regulated lncRNA in lung cancer. Our study fits the following working model. METTL3 is overexpressed by a yet-to-be identified mechanism, which then stabilizes LCAT3 via m6A modification. Overexpressed LCAT3 then recruit FUBP1 to c-MYC promoter to transactivate c-MYC expression, leading to enhanced proliferation, survival, migration/invasion and metastasis of lung cancer cells (Fig. 8G). Our study revealed an oncogenic role of the LCAT3-FUBP1-cMYC axis, and characterize it as a promising prognostic biomarker and a potential therapeutic target for lung cancer.

## Supplementary Information

**Additional file 1:** **Table S1**: Sequences of primers, sgRNAs, siRNA, and shRNA used in the study. **Table S2**: Information of antibodies used in the study. **Table S3**: Quality control metrics of the RNA-seq libraries. **Table S4**: Quality control metrics of the MeRIP-seq libraries. **Table S5**: Proteins identified by Mass Spectrometry analysis of RNA pull-down fractions. **Figure S1**: The full sequence of LCAT3 was confirmed by 3’ RACE and 5’ RACE. (**A**) 3’ RACE assay of LCAT3. (**B**) 5’ RACE assay of LCAT3. (**C**) Full sequence of LCAT3 determined by Sanger sequencing. **Figure S2**: LCAT3 is upregulated by METTL3 through m6A modification. (**A**) No significant difference in LCAT3 CNV between LUAD tumor and noncancerous normal tissues in TCGA. (**B**) No significant association of LCAT3 CNV with its expression in LUAD tumor and noncancerous normal tissues. (**C**) The ENCODE regulation track of UCSC genome browser did not display any regulation elements in LCAT3 locus. (**D**–**F**) Expression of LCAT3 after treatment with 5-Aza-2ʹ-Deoxycytidine (5-Aza-dC) in A549, Calu1 and Hop62 cells at 48 hours and different doses. (**G**) Dot blotting assays detect the m6A modification level in A549 and Calu1 cells. **Figure S3**: Overexpression of LCAT3 promotes proliferation of lung cancer cells. (**A**) qRT-PCR analysis of LCAT3 expression in A549 and Calu1 cells expressing a control empty vector (pcDNA3.1) or LCAT3-OE vector. (**B**) Cell proliferation assays for A549 and Calu1 cells expressing a control empty vector (pcDNA3.1) or LCAT3-OE vector. (**C**) Cell colony formation assays for A549 and Calu1 cells expressing a control empty vector (pcDNA3.1) or LCAT3-OE vector. *P < 0.05; **P < 0.01; ***P < 0.001. **Figure S4**: Stable knockdown of LCAT3 inhibits proliferation, invasion, and migration of lung cancer cells. (**A**) qRT-PCR analysis of LCAT3 expression in Calu1 and Hop62 cells expressing control (Ctrl) or LCAT3 shRNAs. (**B**) Cell proliferation assays for Calu1 and Hop62 cells expressing control (Ctrl) or LCAT3 shRNAs, using CCK8 assay. (**C**, **D**) Transwell invasion and migration assays were performed in stable LCAT3 knockdown and control lung cancer cells. Representative images (left) and statistical analysis (right) are shown. *P < 0.05; **P < 0.01; ***P < 0.001. **Figure S5**: mRNA expression profile of in LCAT3-silenced cells. (**A**) Hierarchical clustering of 1,221 genes that exhibited significantly altered expression in LCAT3-silenced Calu1 cells as compared with vector-transfected control cells. The color bar indicates the fold change (log2). Some cell cycle related genes (CCND1, CCDA2, E2F2, CCNB1, CDK1 and CDK6) were pointed out. (**B**, **C**) qRT-PCR analysis of expression of cell cycle related genes in A549 (**B**) and Calu1 (**C**) cells transfected with LCAT3-siRNA or NC-siRNA. *P < 0.05; **P < 0.01; ***P < 0.001. **Figure S6**: LCAT3 physically interacts with FUBP1. (**A**) Western blotting analysis of FUBP1, DDX5, FUBP3 and HNRPL in sense (S) and antisense (AS) LCAT3 pull-down fractions. GAPDH served as a negative control. (**B**) RNA secondary structure of LCAT3 predicted by RNAfold. The 208–342 nt of LCAT3 forms a stem-loop structure. (**C**) Interaction profile (208–342 nt) of LCAT3 and FUBP1 was predicted by catRAPID. X axis represents the RNA sequence distribution, while Y axis indicates the protein interaction score. The 208–342 nt of LCAT3, which forms a stem-loop structure, is the core sequence binding to FUBP1. (**D**) Calu1 cells were co-transfected with pcDNA3.1-MS2/pcDNA3.1-LCAT3-MS2/pcDNA3.1-208–342 nt-MS2 and MCP-3xFLAG plasmids. FLAG-MCP-MS2 pull-down and western blotting assays were performed to detect binding between LCAT3 and FUBP1. **Figure S7**: LCAT3 and FUBP1 co-regulate the expression of c-Myc. (**A**, **B**) Western blot assays detecting the protein expression of FUBP1 in A549 and Calu1 cells transfected with LCAT3 siRNA (**A**) or LCAT3-OE vector (**B**). (**C**, **D**) qRT-PCR (**C**) and western blot (**D**) analysis of FUBP1 overexpression efficiency in A549 and Calu1 cells expressing a control empty vector (PLVX-IRES-zsGreen1) or FUBP1-OE vector. (**E**) qRT-PCR detecting the mRNA expression of MYC in lung cancer cells with FUBP1 overexpression. (**F**) qRT-PCR detecting the mRNA expression of MYC in lung cancer cells with LCAT3 overexpression. **Figure S8**: LCAT3 has no effect on MYC mRNA stability as well as c-Myc protein stability. (**A**, **C**) Half-life of MYC mRNA in A549 (**A**) and Calu1 (**C**) cells transfected with LCAT3 siRNA or control siRNA. (**B**, **D**) Half-life of c-Myc protein in A549 (**B**) and Calu1 (**D**) cells transfected with LCAT3 siRNA or control siRNA. (**E**, **G**) Half-life of MYC mRNA in A549 (**E**) and Calu1 (**G**) cells transfected with FUBP1 siRNA or control siRNA. (**F**, **H**) Half-life of c-Myc protein in A549 (**F**) and Calu1 (**H**) cells transfected with FUBP1 siRNA or control siRNA. **Figure S9**: LCAT3 recruits FUBP1 to activate MYC expression and regulate expression of its downstream genes. (**A**, **B**, **C**) Fragmented DNA samples were separated by electrophoresis on a 1% agarose gel, corresponding to Figure 8A–8C. The majority of chromatin was digested to 1 to 5 nucleosomes in length (150 to 900 bp). (**D**, **E**) qRT-PCR analysis of expression of MYC downstream genes in A549 (**D**) and Calu1 (**E**) cells transfected with FUBP1-siRNA or NC-siRNA.

## Data Availability

All data generated during this study are included in this published article and its supplementary files.

## Abbreviations

LncRNAs: Long non-coding RNAs
LCAT3: Lung Cancer Associated Transcript 3
FUBP1: Far Upstream Element Binding Protein 1
FUSE: Far-upstream element
CDK: Cyclin-dependent kinase
m6A: N6-methyladenosine
METTL3: Methyltransferase like 3
LUAD: Lung adenocarcinomas
CPAT: Coding Potential Assessment Tool
CPC2: Coding Potential Calculator 2
KEGG: Kyoto Encyclopedia of Genes and Genomes
GO: Gene Ontology
NC: Negative control
RNA-seq: RNA-sequencing
TCGA: The Cancer Genome Atlas
RACE: Rapid amplification of cDNA ends
CCK8: Cell counting kit-8
EdU: Ethynyl deoxyuridine
RIP: RNA immunoprecipitation
CRISPR: Clustered regularly interspaced short palindromic repeats
MeRIP: Methylated RNA immunoprecipitation
ChIP: Chromatin immunoprecipitation
MS: Mass spectrometry
siRNA: Small interfering RNA
shRNA: Short hairpin RNA
SCLC: Small cell lung cancer
NSCLC: Non-small cell lung cancer
CBS: Cystathionine-β-synthase
sgRNAs: Single guide RNAs
HRP: Horseradish peroxidase
FPKM: Transcript per million reads mapped
FDR: False discovery rate
CHX: Cycloheximide
DEGs: Differentially expressed genes
GLCC1: Glycolysis-associated lncRNA of colorectal cancer
UCA1: Urothelial cancer associated 1
DLL4: Delta-like ligand 4
FBPs: FUSE-binding proteins
