# Supplementary material for: LCAT3, a novel m6A-regulated long non-coding RNA, plays an oncogenic role in lung cancer via binding with FUBP1 to activate c-MYC
Source: J Hematol Oncol. 2021 Jul 17;14:112. doi: 10.1186/s13045-021-01123-0 (PMC8285886; doi:10.1186/s13045-021-01123-0)
Supplement: Supplementary file 1 — Additional file 1: Table S1: Sequences of primers, sgRNAs, siRNA, and shRNA used in the study. Table S2: Information of antibodies used in the study. Table S3: Quality control metrics of the RNA-seq libraries. Table S4: Quality control metrics of the MeRIP-seq libraries. Table S5: Proteins identified by Mass Spectrometry analysis of RNA pull-down fractions. Figure S1: The full sequence of LCAT3 was confirmed by 3’ RACE and 5’ RACE. (A) 3’ RACE assay of LCAT3. (B) 5’ RACE assay of LCAT3. (C) Full sequence of LCAT3 determined by Sanger sequencing. Figure S2: LCAT3 is upregulated by METTL3 through m6A modification. (A) No significant difference in LCAT3 CNV between LUAD tumor and noncancerous normal tissues in TCGA. (B) No significant association of LCAT3 CNV with its expression in LUAD tumor and noncancerous normal tissues. (C) The ENCODE regulation track of UCSC genome browser did not display any regulation elements in LCAT3 locus. (D–F) Expression of LCAT3 after treatment with 5-Aza-2ʹ-Deoxycytidine (5-Aza-dC) in A549, Calu1 and Hop62 cells at 48 hours and different doses. (G) Dot blotting assays detect the m6A modification level in A549 and Calu1 cells. Figure S3: Overexpression of LCAT3 promotes proliferation of lung cancer cells. (A) qRT-PCR analysis of LCAT3 expression in A549 and Calu1 cells expressing a control empty vector (pcDNA3.1) or LCAT3-OE vector. (B) Cell proliferation assays for A549 and Calu1 cells expressing a control empty vector (pcDNA3.1) or LCAT3-OE vector. (C) Cell colony formation assays for A549 and Calu1 cells expressing a control empty vector (pcDNA3.1) or LCAT3-OE vector. *P < 0.05; **P < 0.01; ***P < 0.001. Figure S4: Stable knockdown of LCAT3 inhibits proliferation, invasion, and migration of lung cancer cells. (A) qRT-PCR analysis of LCAT3 expression in Calu1 and Hop62 cells expressing control (Ctrl) or LCAT3 shRNAs. (B) Cell proliferation assays for Calu1 and Hop62 cells expressing control (Ctrl) or LCAT3 shRNAs, using CCK8 assay. (C, [file 13045_2021_1123_MOESM1_ESM.pdf]

LCAT3, a novel m6A-regulated long non-coding RNA, plays an oncogenic role in lung cancer via binding with FUBP1 to activate c-MYC

Xinyi Qian, Juze Yang, Qiongzi Qiu, Xufan Li, Chengxi Jiang, Jia Li, Liangliang Dong, Kejing Ying, Bingjian Lu, Enguo Chen, Pengyuan Liu, Yan Lu

Supplementary Data

Table S1 Sequences of primers, sgRNAs, siRNA, and shRNA used in the study

Oligo ids	Sequence (5’→3’)
LCAT3-F	CAGGTCGTAAATGGGAGTTGGA
LCAT3-R	ACTTTCATCACCTTACCAGCGT
FUBP1-F	AATTCGTGGCACTCCACAA
FUBP1-R	ACAGGTGGCCCTAAAGGATT
MYC-F	TGCTCCATGAGGAGACACC
MYC-R	CTTTTCCACAGAAACAACATCG
E2F2-F	TCCTCACAGACAGGCTGACA
E2F2-R	GGCAGCACCTAGTGTCCAAT
CCNA2-F	CCATACCTCAAGTATTTGCCATC
CCNA2-R	TCCAGTCTTTCGTATTAATGATTCAG
CCNB1-F	CATGGTGCACTTTCCTCCTT
CCNB1-R	AGGTAATGTTGTAGAGTTGGTGTCC
CCND1-F	CCTGTCCTACTACCGCCTCA
CCND1-R	CAGTCCGGGTCACACTTGA
CDK1-F	TGGATCTGAAGAAATACTTGGATTCTA
CDK1-R	CAATCCCCTGTAGGATTTGG
CDK6-F	GTGGCCCTCGGAATAGATG
CDK6-R	CAGACAAGAGGAGGCACCAC
β-actin-F	GGACTTCGAGCAAGAGATGG
β-actin-R	AGCACTGTGTTGGCGTACAG
GAPDH-F	AGCTCACTGGCATGGCCTTC
GAPDH-R	CGCCTGCTTCACCACCTTCT
MYC-ChIP-F	GAGGTGGTGGAGGGAGAGAA
MYC-ChIP-R	CCCAGCCCCACACATGATT
LCAT3-3'RACE-1	GGTGTGAGGAGGACTTCATCCTTG
LCAT3-3'RACE-2	CTCACTTGCTCTGTCCCTGGGC
LCAT3-5'RACE-1	CAAGGATGAAGTCCTCCTCACACC
LCAT3-5'RACE-2	GTCACAAGATTGCGGCTTTGTCTGC
pcDNA3.1-LCAT3-F(EcoRI)	TAGTCCAGTGTGGTGGAATTCCTGGTTTAGGCTTTATCGCTGG
pcDNA3.1-LCAT3-R(XhoI)	AACGGGCCCTCTAGACTCGAGCAATTTCAAAACAACACTTTATTAATCTCC
PLVX-IRES-zsGreen1-FUBP1-F(EcoRI)	GGATCTATTTCCGGTGAATTCGCAACCATGGCAGACTATTCAA
PLVX-IRES-zsGreen1-FUBP1-R(BamHI)	GGAGGGAGAGGGGCGGGATCCTTATTGGCCCTGAGGTGCTG
LCAT3-m6A-F	CCGGTTGTACCCTGAGACAG
LCAT3-m6A-R	ACATGGCACTCCCAAGACAC
pGL3-Basic-MYC-F(+33 to -1896bp)	GCGTGCTAGCCCGGGCTCGAGAGCTGCAAACTCAACGGGTAA
pGL3-Basic-MYC-R(+33 to -1896bp)	CAGTACCGGAATGCCAAGCTTCTCTGCCTCTCGCTGGAATTAC
pGL3-Basic-MYC-F(+33 to -1647bp)	GCGTGCTAGCCCGGGCTCGAGCAAATCATGTGTGGGGCTGG
pGL3-Basic-MYC-R(+33 to -1647bp)	CAGTACCGGAATGCCAAGCTTCTCTGCCTCTCGCTGGAATTAC
LCAT3 siRNA2	UUCAUUAGAAGAGCUCUCCTT
LCAT3 siRNA4	UAAACCUCACUCUCACUGCTT
FUBP1 siRNA1	GGAGGAGUUAACGACGCUUTT
FUBP1 siRNA2	GGGACAUCACUGAAUUCAATT
Negative Control siRNA	UUCUCCGAACGUGUCACGUTT
sh1-LCAT3-F	CCGGTTTATGTAGGTTGGAGATTGCCTCGAGGCAATCTCCAACCTACATAAATTTTTG
sh1-LCAT3-R	AATTCAAAAATTTATGTAGGTTGGAGATTGCCTCGAGGCAATCTCCAACCTACATAAA
sh2-LCAT3-F	CCGGCTTTGTACAGTTTGAGTAGGCCTCGAGGCCTACTCAAACTGTACAAAGTTTTTG
sh2-LCAT3-R	AATTCAAAAACTTTGTACAGTTTGAGTAGGCCTCGAGGCCTACTCAAACTGTACAAAG
METTL3-sgRNA#2-F	CACCGGAGTTGATTGAGGTAAAGCG
METTL3-sgRNA#2-R	AAACCGCTTTACCTCAATCAACTCC
METTL3-sgRNA#6-F	CACCGGCTCAACATACCCGTACTAC
METTL3-sgRNA#6-R	AAACGTAGTACGGGTATGTTGAGCC

Table S2 Information of antibodies used in the study

Antibodies	Company	Catalogue#	Species
FUBP1	Proteintech	24864-1-AP	rabbit
c-Myc	Abclonal	A19032	rabbit
METTL3	Proteintech	15073-1-AP	rabbit
Cyclin A2	Cell Signaling	4656	mouse
Cyclin B1	Cell Signaling	12231	mouse
Cyclin D1	Cell Signaling	55506	mouse
GAPDH	Proteintech	60004-1-Ig	mouse

Table S3 Quality control metrics of the RNA-seq libraries

Sample id	Yeild(Gb)	Mean Quality Score	% of >= Q30 Bases	Mapping Rate (%)
siLCAT3-2-1	7.9	38.7	98.4	89.1
siLCAT3-2-2	11.6	38.7	98.3	90.2
siLCAT3-2-3	13.2	38.7	98.4	89.6
siLCAT3-4-1	10.3	38.4	97.8	88.2
siLCAT3-4-2	10.9	38.6	98.2	87.1
siLCAT3-4-3	10.3	38.6	98.3	89.6
siNC-1	9.8	38.7	98.4	90.5
siNC-2	11.1	38.7	98.3	88.3
siNC-3	14.5	38.7	98.4	89.7

Table S4 Quality control metrics of the MeRIP-seq libraries.

Sample ID	Yeild (Gb)	% of >= Q30 Bases	Mean Quality Score	mapping rate (%)
A549-6-7-Input-1	10.8	98.0	38.4	95.3
A549-6-7-Input-2	10.1	98.1	38.5	95.3
A549-6-7-Input-3	10.3	97.8	38.3	94.2
A549-lacZ-Input-1	10.5	97.7	38.3	95.8
A549-lacZ-Input-2	13.8	98.1	38.5	96.3
A549-lacZ-Input-3	9.8	97.6	38.3	95.8
A549-6-7-m6A-1	8.5	96.4	37.6	61.7
A549-6-7-m6A-2	7.5	96.9	37.8	65.1
A549-6-7-m6A-3	8.6	96.4	37.6	52.5
A549-lacZ-m6A-1	10.5	96.1	37.6	68.1
A549-lacZ-m6A-2	9.0	96.1	37.5	78.1
A549-lacZ-m6A-3	8.9	96.7	37.7	65.9

Table S5 Proteins identified by Mass Spectrometry analysis of RNA pull-down fractions

**Gene name**	**Unique peptides**		**Mol. weight [kDa]**
	**AS.**	**Sen.**	
XRCC6	7	18	69.842
MCCB	8	16	61.332
**FUBP1**	**0**	**6**	**67.56**
RBM14	3	6	69.491
IGF2BP2	6	6	66.121
DDX5	0	3	69.147
FUBP3	0	2	61.64
RAVR1	2	2	63.876
RFA1	2	2	68.137
HNRPL	0	2	64.132

Note: Proteins of 60-70 kDd were selected. Proteins that were only present in the sense fraction and had multiple detected peptides are preferentially verified by subsequent western blot and RIP assays.

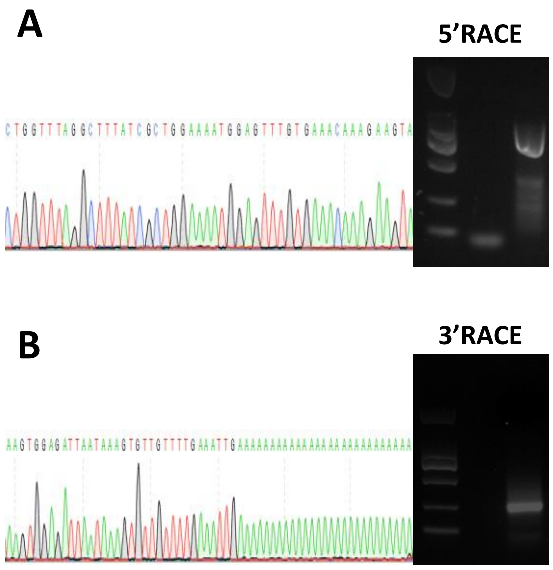

C

Full sequence of LCAT3 (1541nt)

CTGGTTTAGGCTTTATCGCTGGAAAATGGAGTTTGTGAAACAAAGAAGTAATAGATGCATAGCAAGAGCTCA
GTGTCCTTCCACATTGTGTCACCGGTTGTACCCTGAGACAGATTCTCCAAGTCTTAGAACATGGATATGCAGT
GAGAGTGAGGTTTACGTAGAAAAGTCTTTGTGTCTTGGGAGTGCCATGTGTATTGAATGTCTATGACTCATGT
CTCCCCTTGACTCACTTCTTGGCTGGGCTACATGACTCATCAGGCCTGGTAGGTTCAGGGCAGTGTAGCTGG
TGGGGAAGGGCTAAGTGAAGAGGGGCTGTCTAGACCTGGGGAGCATGGTTATAATGAGCTTTTTCAAACGT
ACAACTCAACTCTCTTGCACCTAGGAAGTATCTTCATCCAGAAGGAGTTCTCAGTCCTTTTTCTGGTCCTGCA
GAGCAAAAGAGGAGAGAGAAAAAGAGAATGAGTTACATGGGCCCACTGTATGTTATAGCTGACTGCTGATG
GTATCAGAGATTTTAACACTGCAGTCAACTGCAGACAAAGCCGCAATCTTGTGACTTCACAGAGCCTCATGG
GCTTTTGAGTTGACCAACTTGAGAAAAATTTCTGACTTGATGCATGAAATGAAACGAAGGCAGGTGACACA
TAAAGGAAATGACAAGACACGATTGAAAACTATTGACCCTCAAGTTATCACAAATGGTTATGAAATGGAAAT
GGAATGGGAAGGAGTATGAAAAAATAAGCTGAATACTGACAAACCTAAGAATTATTTCTTCAGCTTTGTCT
TCCAAAGGACAATGGTGTGAGGAGGACTTCATCCTTGTGTAACACCCTGTGTGTGTGTTTTTCTTCTTTCTCT
CACCAAAGCATTTTTTTTTCCTCCCTTTTGCTGGAGGCCTGTCTCTTTCTCTTGAACAGATCAACAATCAGGT
CGTAAATGGGAGTTGGAGCCGGATGATCAAAAAGAACTTTAGAAAAATGGGAATAAAAGCAAGAGCTGAT
GAGTTTGAAATGAAAGCCCATCTGGGAGAGCTCTTCTAATGAACTCAGCCTACTCAAACTGTACAAAGAAA
GCTGAAGAATGATTGCAGCTGCAATCTCCAACCTACATAAAGCACTAACGCTGGTAAGGTGATGAAAGTGAA
GGGAGGTAACTGGTATCTGGATTTGGAAGAGAGAAGTCAAAGGCCATCACCCCCTTGTACTACTGCATTCAC
CATGTAAGTTCCTTCGGAAATTTCACAGCCAATCTGAATTAAAGAGACAGCTGCAGATGCTTCAGGATGAAG
GTCACACTCTGAATTCTACGAAGAGCCCTATGATTCAAAAGTACCATCTCTTTAGCCTCAGAGCAGTTGTATA
CCACCTTCTCACTTGCTCTGTCCCTGGGCTAGTATGGCTTTGAAATTATGTTAAAGAAGAGGCATTTTCCCTC
ACAAAGACTCCCAAAATATGACTATTAAGTGAAAGAACAGGTAGAGGGAGGGAAAGAGCTGAATCTAAGT
GGAGATTAATAAAGTGTTGTTTTGAAATTG

Figure S1. The full sequence of LCAT3 was confirmed by 3’ RACE and 5’ RACE.
(**A**) 3’ RACE assay of LCAT3.
(**B**) 5’ RACE assay of LCAT3.
(**C**) Full sequence of LCAT3 determined by Sanger sequencing.

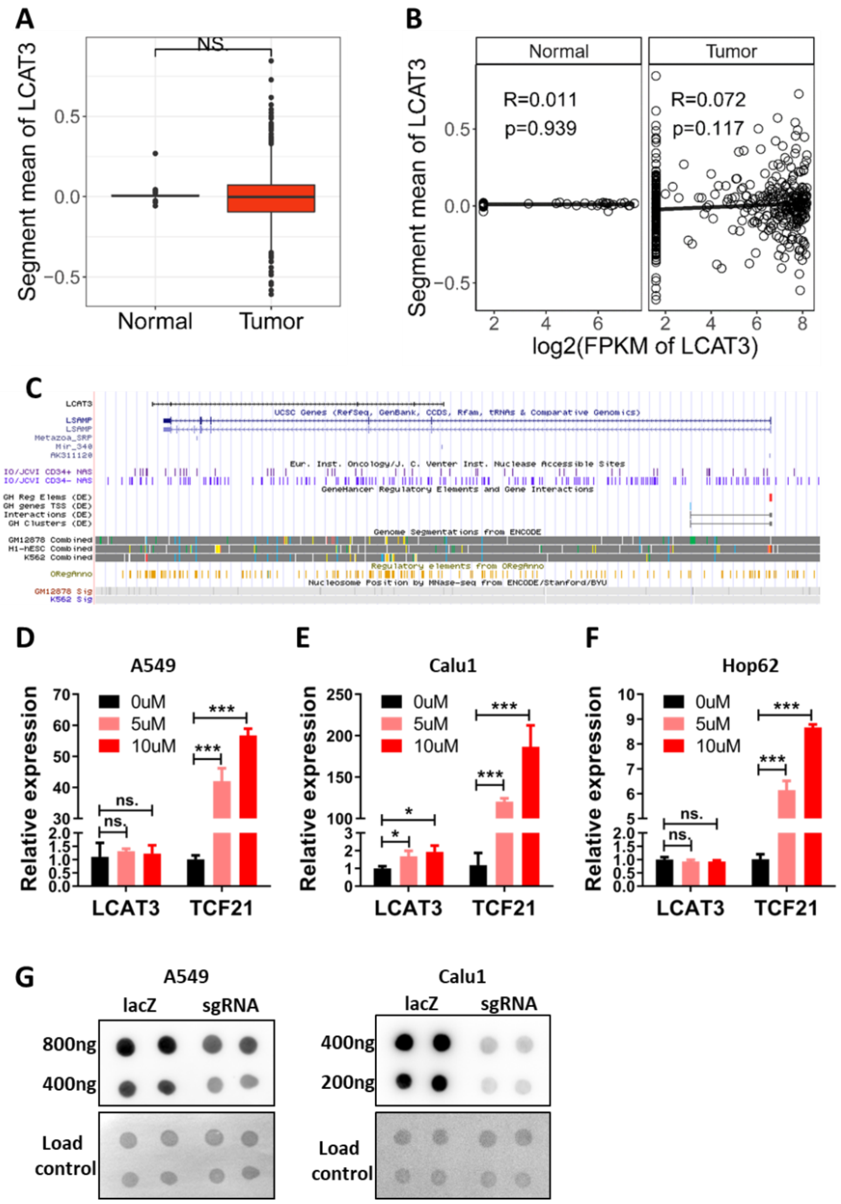

Figure S2. LCAT3 is upregulated by METTL3 through m6A modification.

(**A**) No significant difference in LCAT3 CNV between LUAD tumor and noncancerous normal tissues in TCGA.

(**B**) No significant association of LCAT3 CNV with its expression in LUAD tumor and noncancerous normal tissues.

(**C**) The ENCODE regulation track of UCSC genome browser did not display any regulation elements in LCAT3 locus.

(**D-F**) Expression of LCAT3 after treatment with 5-Aza-2′-Deoxycytidine (5-Aza-dC) in A549, Calu1 and Hop62 cells at 48 hours and different doses.

(**G**) Dot blotting assays detect the m6A modification level in A549 and Calu1 cells.

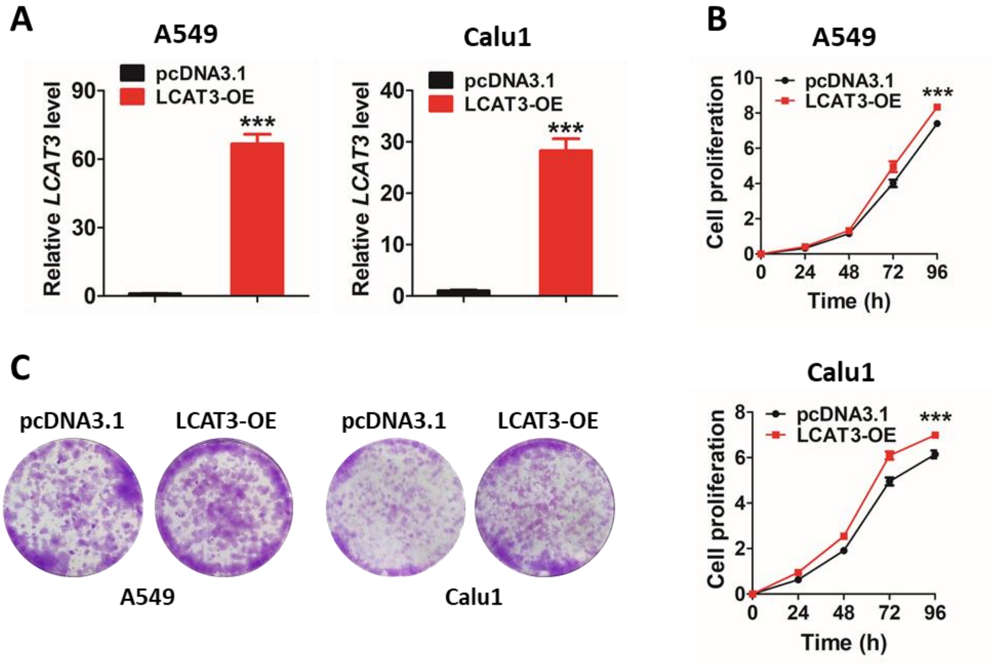

Figure S3. Overexpression of LCAT3 promotes proliferation of lung cancer cells.

(**A**) qRT-PCR analysis of LCAT3 expression in A549 and Calu1 cells expressing a control empty vector (pcDNA3.1) or LCAT3-OE vector.

(**B**) Cell proliferation assays for A549 and Calu1 cells expressing a control empty vector (pcDNA3.1) or LCAT3-OE vector.

(**C**) Cell colony formation assays for A549 and Calu1 cells expressing a control empty vector (pcDNA3.1) or LCAT3-OE vector.

*, $P<0.05$; **, $P<0.01$; ***, $P<0.001$.

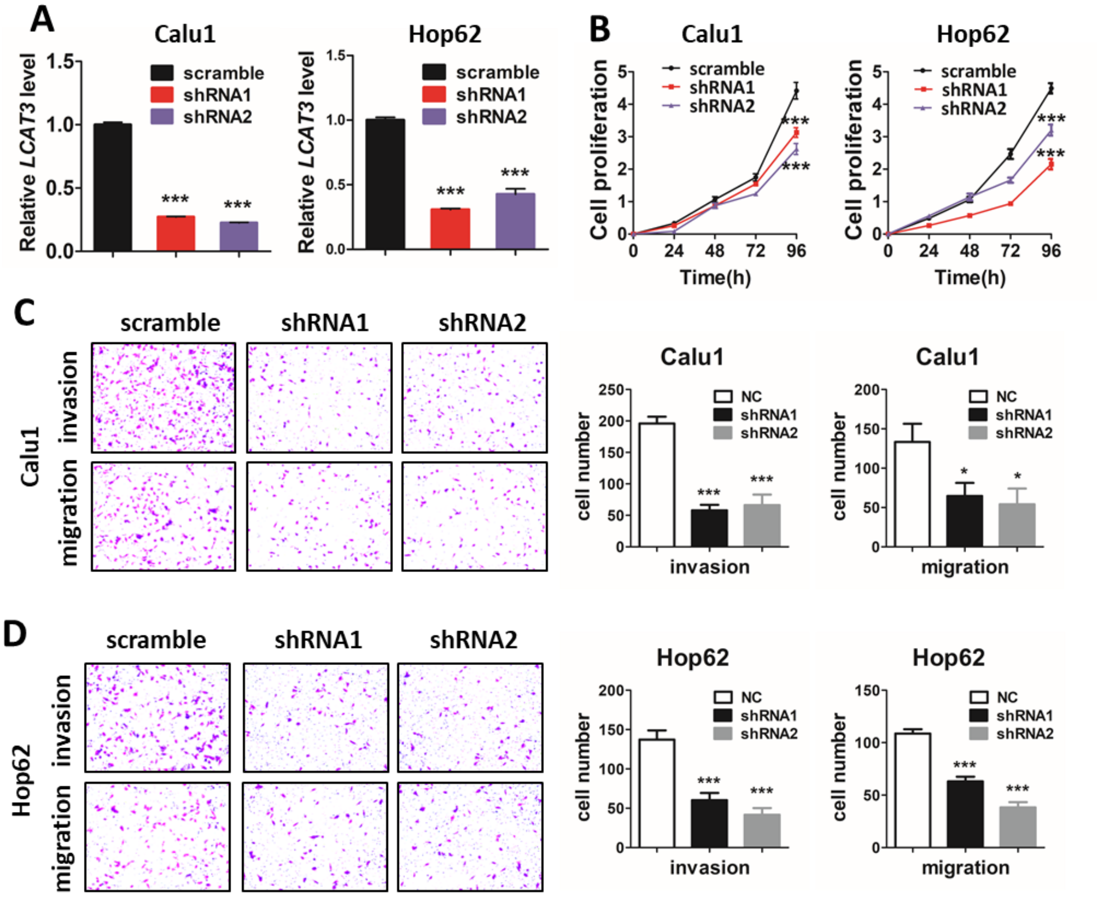

Figure S4. Stable knockdown of LCAT3 inhibits proliferation, invasion, and migration of lung cancer cells.

(**A**) qRT-PCR analysis of LCAT3 expression in Calu1 and Hop62 cells expressing control (Ctrl) or LCAT3 shRNAs.

(**B**) Cell proliferation assays for Calu1 and Hop62 cells expressing control (Ctrl) or LCAT3 shRNAs, using CCK8 assay.

(**C, D**) Transwell invasion and migration assays were performed in stable LCAT3 knockdown and control lung cancer cells. Representative images (left) and statistical analysis (right) are shown.

*, $P<0.05$; **, $P<0.01$; ***, $P<0.001$.

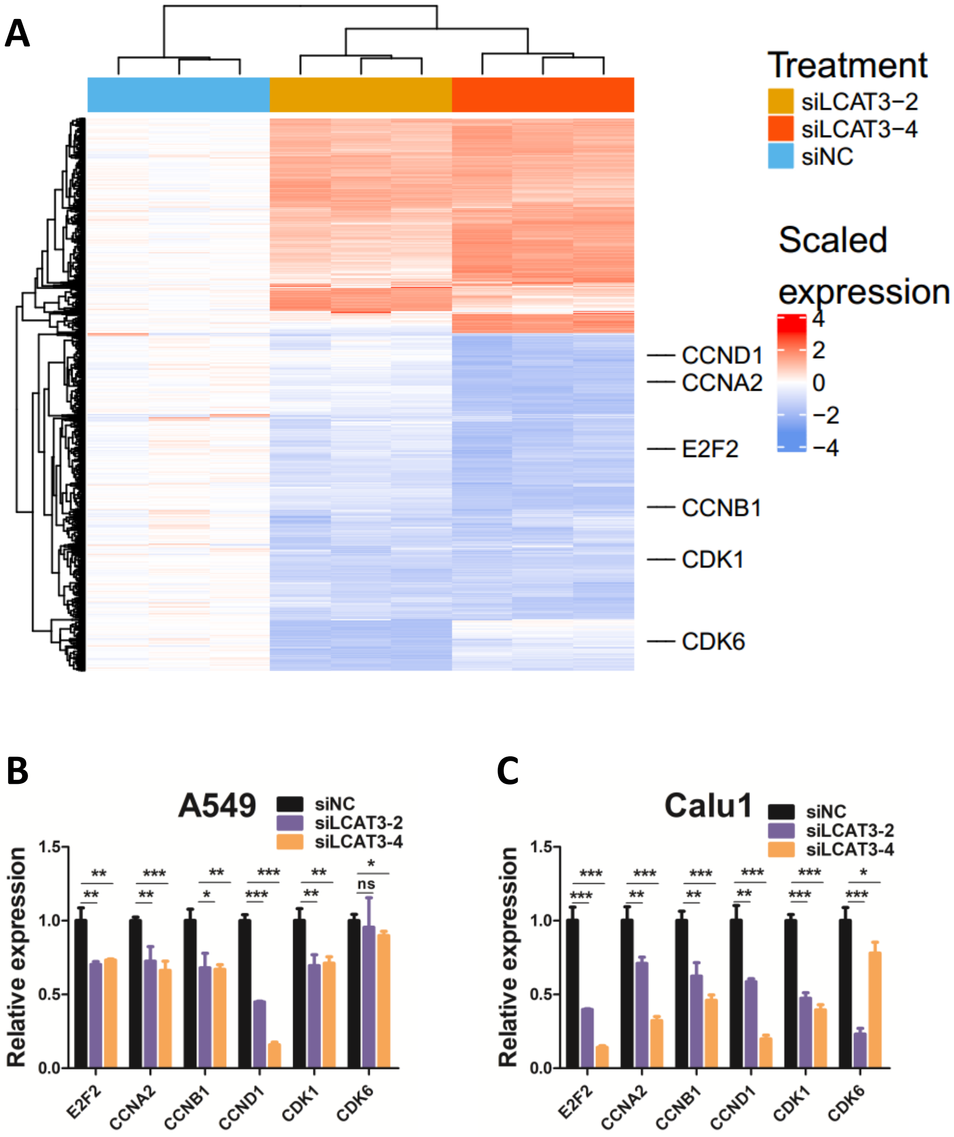

Figure S5. mRNA expression profile of in LCAT3-silenced cells. **(A)** Hierarchical clustering of 1,221 genes that exhibited significantly altered expression in LCAT3-silenced Calu1 cells as compared with vector-transfected control cells. The color bar indicates the fold change (log2). Some cell cycle related genes (CCND1, CCDA2, E2F2, CCNB1, CDK1 and CDK6) were pointed out. (**B, C**) qRT-PCR analysis of expression of cell cycle related genes in A549 (**B**) and Calu1 (**C**) cells transfected with LCAT3-siRNA or NC-siRNA.
*, P<0.05; **, P<0.01; ***, P<0.001.

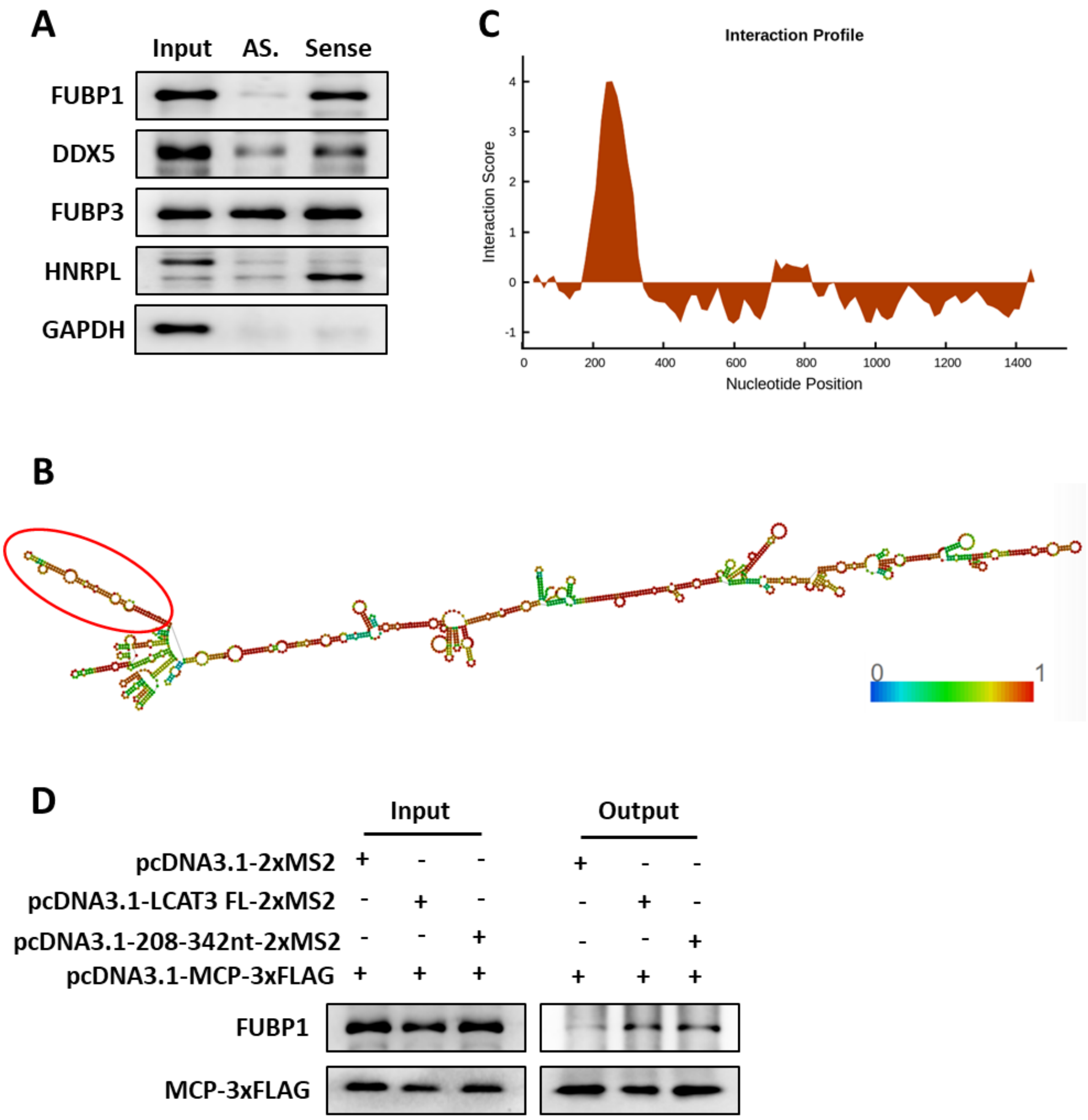

Figure S6. LCAT3 physically interacts with FUBP1.

(**A**) Western blotting analysis of FUBP1, DDX5, FUBP3 and HNRPL in sense (S) and antisense (AS) LCAT3 pull-down fractions. GAPDH served as a negative control.

(**B**) RNA secondary structure of LCAT3 predicted by RNAfold. The 208-342nt of LCAT3 forms a stem-loop structure.

(**C**) Interaction profile of LCAT3 and FUBP1 (208-342 nt) was predicted by catRAPID. X axis represents the RNA sequence distribution, while Y axis indicates the protein interaction score. The 208-342nt of LCAT3, which forms a stem-loop structure, is the core sequence binding to FUBP1.

(**D**) Calu1 cells were co-transfected with pcDNA3.1-MS2/pcDNA3.1-LCAT3-MS2/pcDNA3.1-208-342 nt-MS2 and MCP-3xFLAG plasmids. FLAG-MCP-MS2 pull-down and western blotting assays were performed to detect binding between LCAT3 and FUBP1.

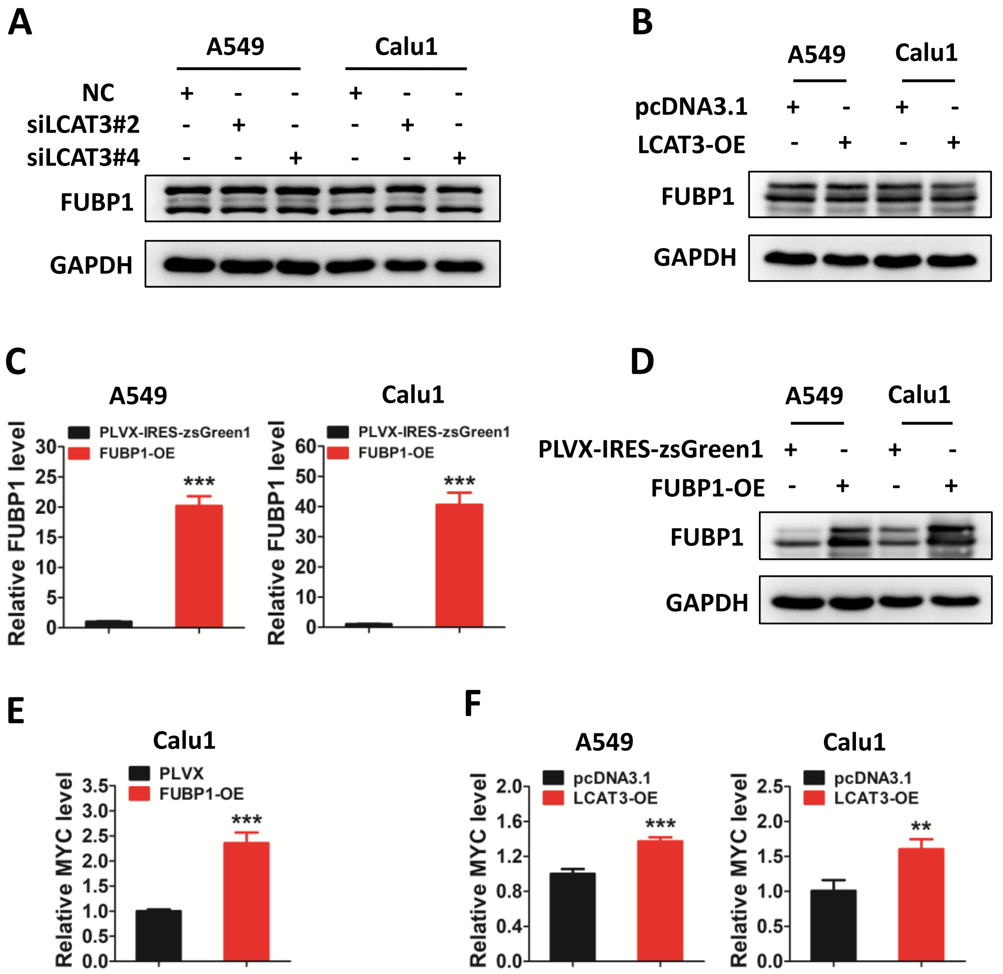

Figure S7. LCAT3 and FUBP1 co-regulate the expression of c-Myc.

(**A, B**) Western blot assays detecting the protein expression of FUBP1 in A549 and Calu1 cells transfected with LCAT3 siRNA (**A**) or LCAT3-OE vector (**B**).

(**C, D**) qRT-PCR (**C**) and western blot (**D**) analysis of FUBP1 overexpression efficiency in A549 and Calu1 cells expressing a control empty vector (PLVX-IRES-zsGreen1) or FUBP1-OE vector.

(**E**) qRT-PCR detecting the mRNA expression of MYC in lung cancer cells with FUBP1 overexpression.

(**F**) qRT-PCR detecting the mRNA expression of MYC in lung cancer cells with LCAT3 overexpression.

*, P<0.05; **, P<0.01; ***, P<0.001.

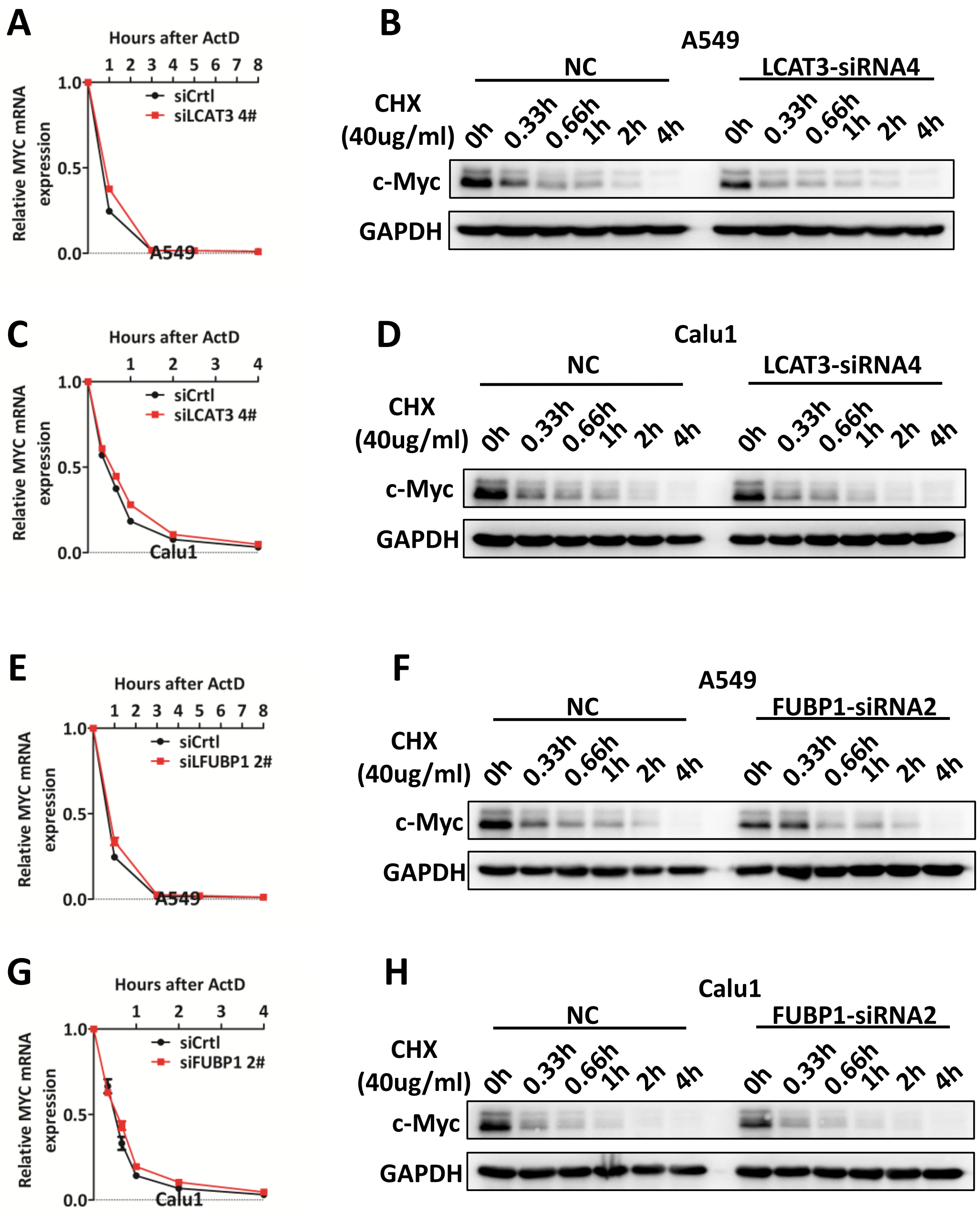

Figure S8. LCAT3 has no effect on MYC mRNA stability as well as c-Myc protein stability.
(**A, C**). Half-life of MYC mRNA in A549 (**A**) and Calu1 (**C**) cells transfected with LCAT3 siRNA or control siRNA.
(**B, D**). Half-life of c-Myc protein in A549 (**B**) and Calu1 (**D**) cells transfected with LCAT3 siRNA or control siRNA.
(**E, G**) Half-life of MYC mRNA in A549 (**E**) and Calu1 (**G**) cells transfected with FUBP1 siRNA or control siRNA.
(**F, H**) Half-life of c-Myc protein in A549 (**F**) and Calu1 (**H**) cells transfected with FUBP1 siRNA or control siRNA.

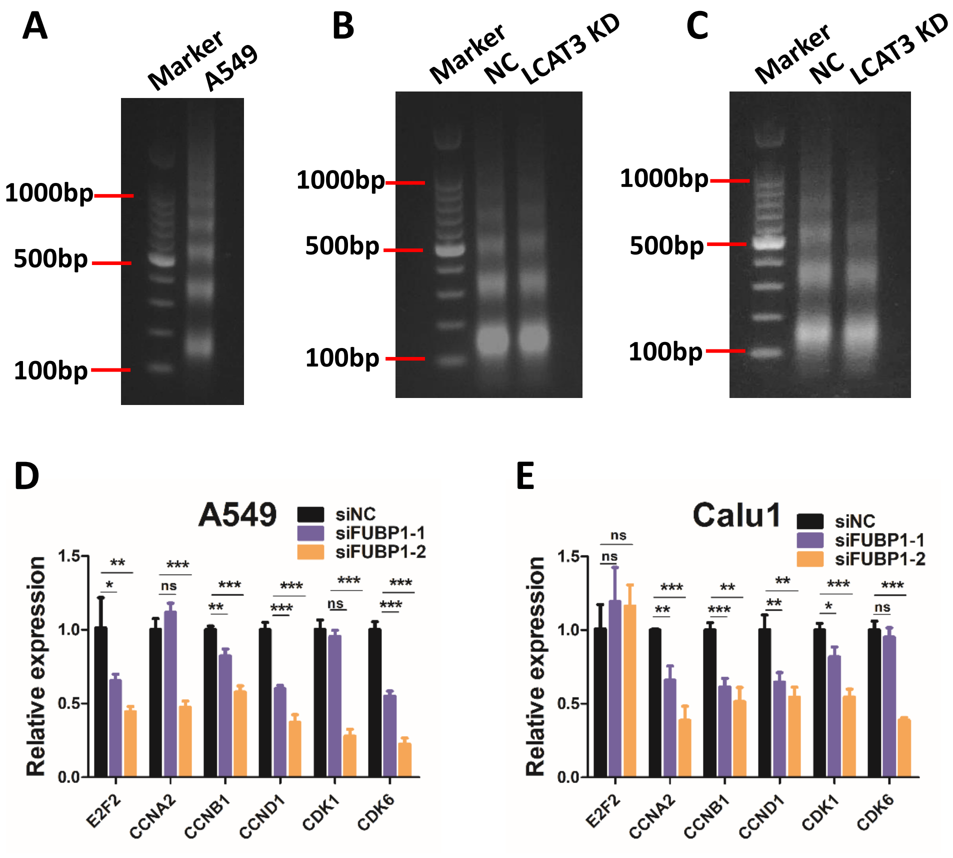

Figure S9. LCAT3 recruits FUBP1 to activate MYC expression and regulate expression of its downstream genes.

(**A, B, C**) Fragmented DNA samples were separated by electrophoresis on a 1% agarose gel, corresponding to Figure 8A–8C. The majority of chromatin was digested to 1 to 5 nucleosomes in length (150 to 900 bp).

(**D, E**) qRT-PCR analysis of expression of MYC downstream genes in A549 (**D**) and Calu1 (**E**) cells transfected with FUBP1-siRNA or NC-siRNA.

*, P<0.05; **, P<0.01; ***, P<0.001.
